# Advanced maternal age increases the risk of adverse neonatal outcomes: a comparative study in Ethiopia

**DOI:** 10.1186/s12884-025-08316-2

**Published:** 2025-10-28

**Authors:** Yonas Mengistu Abebe, Luel Deribe, Addishiwot Fantahun, Belda Negesa Beyene, Solomon Medina Megule, Wondimagegn Genaneh Shiferaw, Kebede Feyisa Adugna, Lewegneh Wegayehu Tessema, Alemwork Abie Getu, Eden Asmare Kassahun, Bezawit Abeje Alemayehu, Amanuel Tebabal Nega, Zigijit Azene, Simachew Animen Bante, Amlaku Mulat Awoke, Fentahun Alemnew Chekole, Wondu Feyisa Balcha

**Affiliations:** 1 Saint Paulo’s Hospital, 1271, Addis Ababa, Ethiopia; 2 Department of Midwifery, School of Nursing and Midwifery, College of Medicine and Health Sciences, Addis Ababa University, 9086, Addis Ababa, Ethiopia; 3 Department of Midwifery, Institute of Health, Bule Hora University, 144, Bule Hora, Ethiopia; 4 Department of Nursing, College of Health Sciences, Bonga University, 334, Bonga, Ethiopia; 5 Department of Emergency and Critical Care Nursing, College of Health Sciences and Medicine, Wolaita Sodo University, 138, Wolaita Sodo, Ethiopia; 6 Department of Pharmaceutics, College of Medicine and Health Sciences, Bahir Dar University, 079, Bahir Dar City, Ethiopia; 7 Department of Midwifery, Gambella Teachers and Health Science College, Gambella, Ethiopia; 8 Department of Midwifery, College of Medicine and Health Sciences, Bahir Dar University, 079, Bahir Dar, Ethiopia

**Keywords:** Adult, Advanced maternal age, Adverse neonatal outcomes, Pregnancy, Unfavorable outcomes, Mothers, Neonates, Preterm birth, Low birth weight, Stillbirth, Addis ababa, Ethiopia

## Abstract

**Background:**

Adverse neonatal outcomes (ANOs), including preterm birth, low birth weight, low Apgar scores, congenital malformations, and early neonatal death, remain a leading cause of neonatal and infant mortality worldwide. Pregnancy at advanced maternal age (> 35 years) increases the risk of ANOs, even in the absence of other socio-economic or medical risk factors. In developing countries such as Ethiopia, where neonatal mortality is high, evidence on the impact of advanced maternal age on neonatal outcomes is limited. This highlights the need for context-specific research to inform maternal and neonatal health interventions.

**Objective:**

This study aimed to compare adverse neonatal outcomes and associated factors among adult and advanced-aged pregnant mothers at the public hospitals of Addis Ababa City.

**Methods:**

A hospital-based comparative cross-sectional study was conducted at public hospitals in Addis Ababa from April 29 to June 30, 2024, among 707 mothers (471 adults and 236 advanced-age). Data were collected using a systematic random sampling technique and the Open Data Kit (ODK) Collect Android application. Analyses were performed with SPSS version 26.0. Independent t-tests were used to compare continuous outcome variables between groups and estimate mean differences, while binary logistic regression analyses were employed to estimate COR and AOR with a CI of 95%. A *p*-value < 0.05 was considered statistically significant.

**Results:**

The overall response rate was 98.6%. Nearly half of advanced-aged mothers (51.5%; 95% CI: 44.7–57.9) experienced at least one ANO, compared to 40.2% (95% CI: 35.7–44.6) among adult-aged mothers, yielding a proportion difference of 11.3% (95% CI: 3.5–19.1). Overall, 44.0% (95% CI: 40.2–47.6) of neonates experienced at least one ANO. Advanced maternal age was significantly associated with unfavorable neonatal outcomes [AOR = 1.51; 95% CI: 1.02–2.25]. Specifically, advanced age was linked to increased risks of preterm birth [AOR = 1.84; 95% CI: 1.18–2.85] and large for gestational age [AOR = 2.68; 95% CI: 1.31–5.49], while it was inversely associated with post-term birth [AOR = 0.25; 95% CI: 0.08–0.83].

**Conclusions:**

Advanced maternal age was significantly associated with increased ANOs, particularly preterm birth, and large for gestational age. To mitigate these risks, there is a need for strategy development that creates awareness on the advantages of achieving the desired number of children during early adulthood, alongside strengthening risk-based antenatal care in Ethiopia.

**Supplementary Information:**

The online version contains supplementary material available at 10.1186/s12884-025-08316-2.

## Introduction

Adverse neonatal outcomes (ANOs) refer to unfavorable health events that occur in the newborn during the first 28 days of life [1, 2]. Core ANOs commonly reported in studies include neonatal death, classified as early (within 7 days) or late (8–28 days) [3, 4]; preterm birth, defined as delivery before 37 completed weeks of gestation, is further categorized into very preterm (28–<32 weeks), moderate preterm (32–<34 weeks), and late preterm (34–<37 weeks) [5], weight-related outcomes such as low birth weight (LBW) (< 2,500 g), small for gestational age (SGA) (< 10th percentile), and large for gestational age (LGA) (>90th percentile) [6], and a low Apgar score at 5 min (< 7), which predicts poor neonatal outcomes [3, 7]. Extended ANOs often assessed in epidemiological studies include postterm birth [8], neonatal intensive care unit (NICU) admission [9]; congenital malformations like neural tube defects (NTDs) [10, 11]; neonatal infections such as sepsis, pneumonia, and meningitis) [12]; intrauterine growth restriction (IUGR) [9, 13], jaundice [14], and birth trauma (e.g., fractures, nerve injuries), which are frequently used as markers of severe morbidity [15].

Adverse neonatal outcomes are key indicators of neonatal health and are influenced by maternal, perinatal, and healthcare-related factors, reflecting the quality of maternal and newborn care [16]. Among these factors, advanced maternal age (AMA) (≥ 35 years) has been identified as an independent and substantial risk factor for ANOs [17–20]. Pregnancy in AMA is defined as a pregnancy in which the estimated delivery date falls when the mother is ≥ 35 years of age [21]. The pregnancy rate among women of AMA is increasing worldwide [22, 23], ranging from 20 to 40% in developed countries [22, 24]. In Ethiopia, the prevalence of giving birth at AMA was varies regionally, with the 2019 Ethiopian Demographic and Health Survey (DHS) reporting approximately 12.7% nationally [25]. This increase is attributed to the growing population of women aged ≥ 35 years [26], and social and cultural shifts influencing women’s reproductive choices, including delaying pregnancy for economic reasons or due to remarriage [27].

Advanced maternal age is associated with increased perinatal morbidity, including low birth weight, preterm birth, low Apgar score, SGA, IUGR, LGA, NICU admission, congenital malformations, and neonatal mortality, including stillbirth [10, 11, 17, 28–31]. These outcomes have long-term impacts on individuals, families, and society [32]. Studies from high-income countries report preterm birth among AMA women ranging from 7% to 48% [18–24, 33]. In Ethiopia, 30–35% of AMA mothers may experience adverse neonatal outcomes compared to 15–20% among adult-aged mothers [34–39]. Globally, preterm birth causes over one million neonatal deaths annually, with 15 million infants born before 37 weeks each year, and LBW accounts for 15–20% of all births [5, 40, 41]. In Ethiopia, a systematic review reported a preterm birth prevalence of 10.5%, regardless of maternal age [42], and confirmed that AMA increases the risk of perinatal mortality [17].

**Fig. 1:**
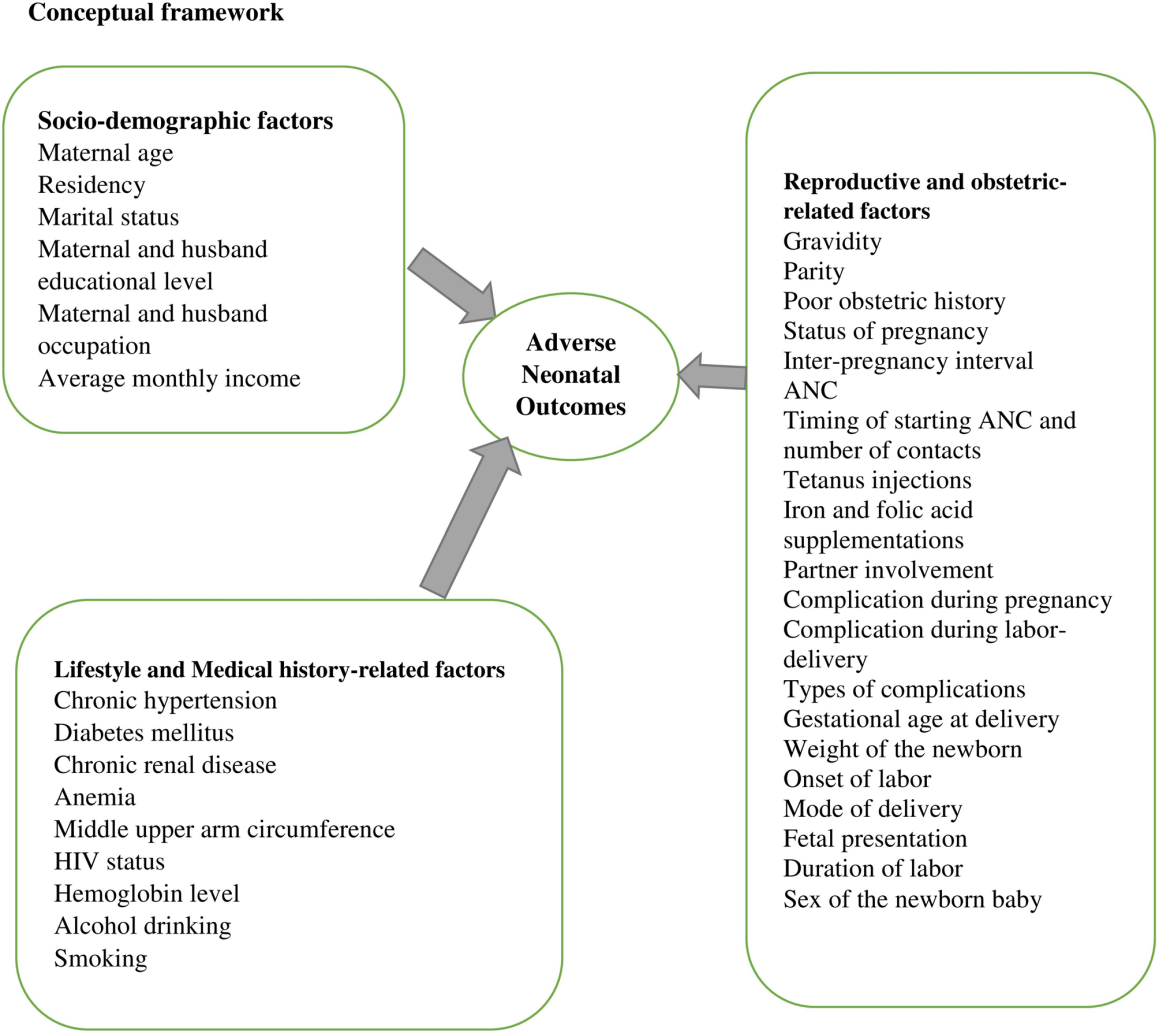
Conceptual framework adapted from different works of literature

Women of AMA face higher risks of complications, including overweight, gestational diabetes, pregnancy-induced hypertension, malpresentation, placental problems, maternal near-miss events, postpartum hemorrhage, premature rupture of membranes, induced labor, and assisted delivery, all of which increase the likelihood of ANOs [18, 30, 34, 43]. Evidence from South Africa, Tanzania, Nigeria, Zambia, and Egypt shows that ANOs, such as stillbirths, are more frequent with extreme maternal ages (< 20 and ≥ 35 years), multiparity, short birth intervals, bad obstetric history (BOH), abnormal presentation, placental and amniotic fluid complications, anemia, malaria, poverty, rural residency, and inadequate antenatal care, as well as neonatal issues like asphyxia and meconium aspiration syndrome [44–48]. Similarly, studies from Ethiopia report that AMA, unfavorable maternal socio-demographic conditions, antepartum and intrapartum complications, and poor maternal nutritional status, such as mid-upper arm circumference (MUAC) < 23 cm, are linked to higher risk of ANOs (Fig. 1) [34–39].

Pregnancy at AMA increases the risk of ANOs, which contribute to neonatal mortality and reflect the burden of neonatal morbidity, particularly in low- and middle-income countries where limited access to skilled care and delayed interventions worsen outcomes, potentially hindering progress toward Sustainable Development Goal (SDG) targets [16]. In Ethiopia, AMA has been linked to higher rates of preterm birth, LBW, and low Apgar scores [34–39]. Existing Ethiopian studies have limitations, as most reported ANOs with adverse perinatal outcomes or use secondary data, limiting causal inferences [37, 38]. Evidence also lacks regional diversity and comprehensive analysis of maternal, obstetric, and socio-economic factors. Updated research is needed to inform interventions and reduce neonatal mortality toward the SDG target of < 12 per 1,000 live births by 2030 [49]. This study aims to compare the prevalence of ANOs and associated factors among adult and AMA pregnancies in public hospitals of Addis Ababa, Ethiopia. Findings will inform interventions to improve neonatal health outcomes and guide maternal and child health policy.

## Methods and materials

### Study area

The study was conducted at selected public hospitals in Addis Ababa City. Addis Ababa is the capital city of Ethiopia and covers an area of 527 km. It is located on a well-watered plateau surrounded by hills and mountains, in the geographic center of the country. There are 13 public hospitals in the city and except Ammanuel Mental Health Hospital, 12 of them give maternal and MCH services, of these five hospitals are administered under the Federal Ministry of Health, six are managed by the Addis Ababa Health Bureau, one is under the police force, and one is governed by the armed force. The study will be conducted at Saint Paulo’s Hospital (SPH), Gandhi Memorial Hospital, (GMH), Minilik Specialize Hospital (MSH), Tikur Anbesa Specialized Hospital (TASH), Yekatit 12 Hospital (Y12H), Petros Hospital (PsH). Each of these hospitals provides inpatient and outpatient services and has MCH services including FP, ANC, labor and delivery, postnatal care, child immunization, and other services. The labor and childbirth services provided in these hospitals are free for all women like the rest of public health facilities in Ethiopia.

### Study design and period

A hospital-based comparative cross-sectional study was conducted from April 29/2024 to June 30/2024.

### Source population

Exposed: All advanced-age mothers (AAMs; >35 years) who gave birth at ≥ 28 weeks of gestation in the selected public hospitals of Addis Ababa City.

Unexposed: All adult-aged mothers (20–34 years) who gave birth at ≥ 28 weeks of gestation in the selected public hospitals of Addis Ababa City.

### Study population

Exposed: Systematically selected AAMs (> 35 years) who gave birth at ≥ 28 weeks of gestation in the selected public hospitals of Addis Ababa City during the data collection period.

Unexposed: Systematically selected adult-aged mothers (20–34 years) who gave birth at ≥ 28 weeks of gestation in the selected public hospitals of Addis Ababa City during the data collection period.

### Inclusion criteria

All mothers aged ≥ 20 years who gave birth at ≥ 28 weeks of gestation at the public hospitals of Addis Ababa City were included in this study.

### Exclusion criteria

Mothers with unknown or unreliable last normal menstrual periods (LNMP) or without early obstetric ultrasound, as well as those with stillbirths or multiple pregnancies, were excluded from the study.

### Sample size determination

The sample size was calculated using a double population formula by considering the following assumptions: taking the proportion of ANOs from a study conducted in Northwest Ethiopia, which was found that the proportion (P1) among AAMs (exposed) was 40.5% and the proportion (P2) among adult aged mothers (unexposed) was 29.4% [37], with a 95% of the level of confidence and a power of 80% calculated as follows:

$$n_1=\left[\underline{(z\alpha/2\sqrt(\overline{p}\overline{q}1+1/\lambda))+z\upbeta\sqrt(p_1q_1+p_2q_2/\lambda))}/\Delta\right]^2$$

Where: $$\mathrm{n}_2=\mathrm{n}_1\lambda,\mathrm{\overline{p}}=(\mathrm{p}+\lambda\mathrm{p}_2)/(1+\lambda),\mathrm{\overline{q}}=1-\mathrm{\overline{p}},\Delta=|\mathrm{p}1-\mathrm{p}2|,\lambda=\mathrm{n}2/\mathrm{n}1$$.

Zα/2 = Value of Z for the level of significance alpha (at 0.05 level of significance value of Z is 1.96), Zβ = power, which indicates that change did not occur by chance. Value of Z for power β (at power level 0.80, the value of Zβ is 0.84).

P1 = proportion of ANOs among AAMs (exposed) = 0.405 [37].

P2 = proportion of ANOs among adult-aged mothers (unexposed) = 0.294 [37].

Therefore, p̄ = (0.405 + 2*0.294)/1 + 2 = 0.33, q̄ = 1-0.33.33 = 0.67, q1 = 1-0.405.405 = 0.595, q2 = 1-0.294.294 = 0.706, ∆ = 0.405 − 0.294 = 0.111, and λ = n2/n1 = 2 at a ratio of 1:2 for AAMs (exposed) and adult aged mothers (unexposed) respectively.

$$\textbf{n1}=\frac{\left[1.96\sqrt{0.33}\;\ast\;0.67\left(1\;+1/2\right)+0.84\sqrt{0.405}\;\ast\;0.595\;+\;0.294\;\ast\;{0.706/2}\right]^2=214}{\left(0.111\right)^2}$$

**n2** = 2*214 = 428. By adding a 10% non-response rate the sample size becomes (214 + 214*10/100 = 236 for AAMs, and 428 + 428*10/100 = 471 for adult-aged mothers), which gives a total sample size of 236 + 471 = 707. Therefore, the final sample size becomes 707 women (236 AAMs and 471 adult-aged mothers).

### Sampling procedure and sampling technique

In Addis Ababa, 12 public hospitals give MCH care services. From these public hospitals in the city, six hospitals were selected by a simple random (lottery) method. The selected hospitals were SPH, GMH, MSH, TASH, Y12H and PsH. Then the two-month delivery report was taken from each selected hospital. From January 1/2024 to February 30/2024 a total of 1230 (780 adults and 455 AAMs) women gave childbirth in selected hospitals; 270 (160 adults and 110 AAMs) in SPH, 375 (225 adults and 150 AAMs) in GMH, 140 (94 adult and 46 AAMs) in MSH, 180 (115 adult and 65 AAMs) in TASH, 140 (100 adult and 40 AAMs) in Y12H and 130 (86 adult and 44 AAMs) in PsH. The total sample size of the study is proportionally allocated for each hospital and each group (adult and AAMs) based on the two months delivery report as follows:

Sample size in the hospital (ni) = the final sample size (n) * total sample population in the hospital (Ni).

Total population sample of the hospitals (N).

After proportional allocation, the sample size for each hospital became 152 (97 adults and 57 AAMs) for SPH, 214 (136 adults and 78 AAMs) for GMH, 81 (57 adults and 24 AAMs), 103 (69 adults and 33 AAMs) for TASH, 81 (60 adult and 21 AAMs) for Y12H and 75 (52 adult and 23 AAMs) for PsH. The eligible women were invited to participate using a systematic random sampling technique.

**Fig. 2:**
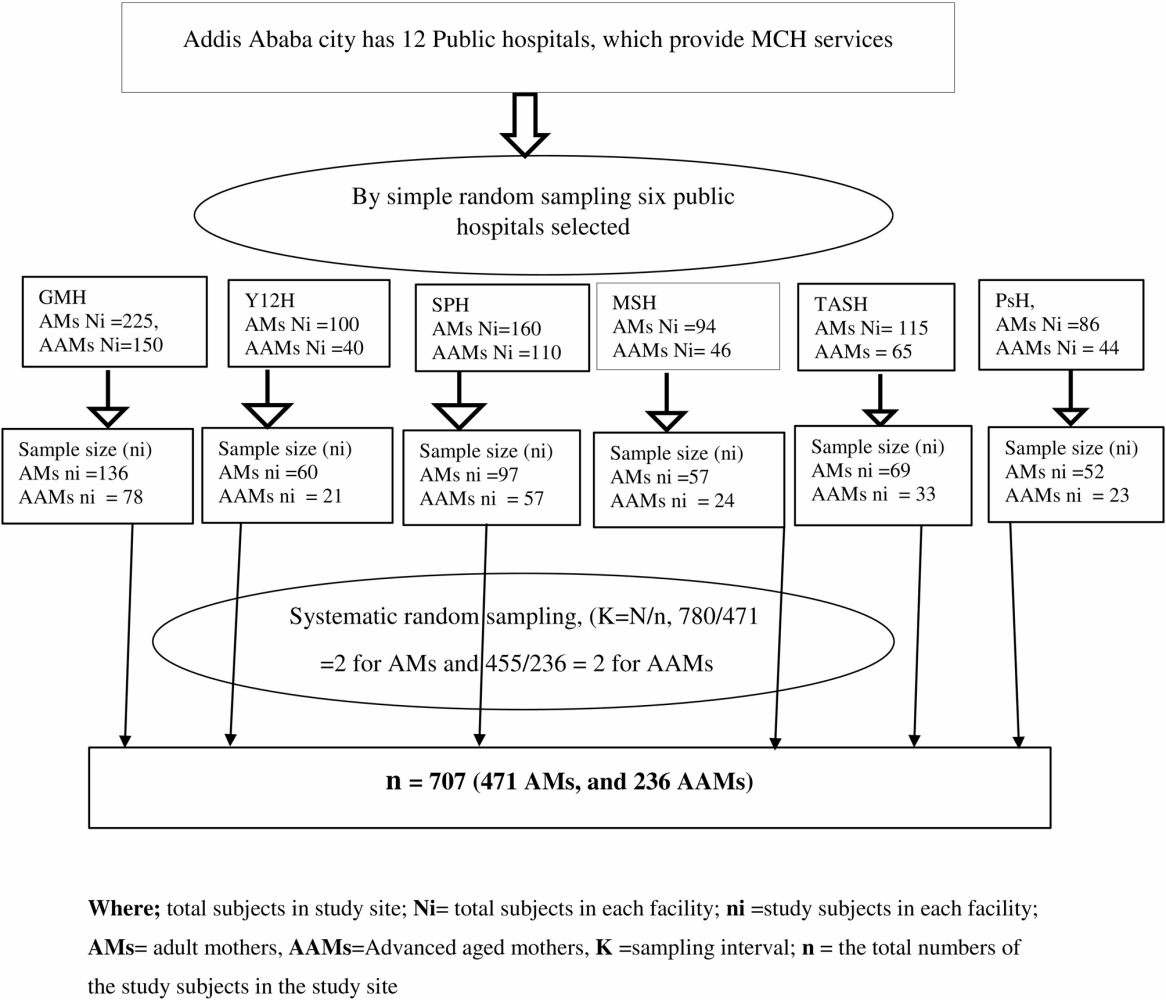
Schematic presentation of sampling procedure

The sampling fraction or K^th^ units was calculated by dividing the total two months deliveries in selected public hospitals by the sample size of the study (780/471 = 2 for adults) and (455/236 = 2 for AAMs). The starting unit was selected by using the lottery method among the first K^th^ units (Fig. 2).

### Dependent variable

Adverse neonatal outcomes.

### Independent variables

- Socio-demographic variables (age, residence, maternal and husband educational level, maternal and husband occupation status, marital status, average monthly income of the family).
- Reproductive and obstetric-related variables (gravidity, parity, bad obstetric history, inter-pregnancy interval, gestational age (GA), ANC contact, timing of starting ANC contact, number of ANC contacts, iron and folic acid supplementation (IFAS), and duration of taking tetanus the supplemented iron and folic acid, TT vaccination, and number of injections, fetal presentation, onset of labor, mode of delivery, indication for C/S, duration of labor, status of pregnancy, complication during pregnancy and labor-delivery, sex of the newborn, Rh status, preeclampsia, PROM, APH, and GDM).
- Lifestyle and medical disease-related (alcohol drinking, smoking, chronic hypertension, pre-pregnancy DM, anemia, chronic renal disease, MUAC, HIV status, hemoglobin level).

### Operational and terms definitions

 Advanced maternal age is considered when maternal age is *≥* 35 years old [21].

 Adult maternal age is considered when maternal age is between 20 and 34 years old [21].

 Adverse neonatal outcome is the occurrence of at least one of the following: LBW, preterm birth, SGA, LGA, LGA/macrosomia, low 5th minute Apgar score, birth injury, congenital malformation, IUGR, NICU admission, neonatal death within 24 h, and post-maturity. The neonate was considered to have ANOs if it experienced at least one of the ANOs [34–39, 50].

 A low 5th -minute Apgar score is when the score is < 7 at the fifth minute of life [51].

 Preterm birth is a birth before 37 completed weeks of GA [5].

 Low birth weight is weighing of newborn less than 2500 g [6].

 Small for Gestational Age is the birth weight of a newborn less than the 10th percentile [6].

 Large for Gestational Age is the birth weight of a newborn greater than the 90th percentile [4].

 Neonatal death is the death of a newborn between 0 and 28 days of life [4].

 A gross congenital anomaly is when the newborn has been diagnosed with a congenital anomaly (hydrocephalus, spinal bifida, anencephaly, cleft lip or pallet, and polydactyl) [10, 11].

 Birth trauma refers to injuries sustained by the newborn during labor and delivery (fractures of the clavicle and humerus, cephalohematoma, intracranial hemorrhage, and brachial plexus palsy [15].

 Bad obstetric history is considered when the woman has at least one of the following conditions in a previous pregnancy: stillbirth, early neonatal death, and *≥* 3 recurrent abortions [52].

Short inter-pregnancy interval is when < 24 months from the date of birth to the conception of the subsequent pregnancy or/and who had an abortion history of < 6 months.

### Data collection tool and procedure

The data was collected using a structured interviewer-administered questionnaire and chart reviews were performed using a checklist by a pre-programmed, ODK Collect Android mobile application. The tool was adapted from relevant works of literature and modified to the local context [34–39, 50]. The questionnaire was first prepared in English language and then translated into Amharic language and back to the English language again to maintain its consistency (supplementary file 1). The questionnaire consisted of socio-demographics, obstetric and reproductive characteristics, medical and lifestyle-related data, and neonatal outcomes-related questions. After assessing eligibility and obtaining informed verbal consent; an exit interview was carried out during the discharge time in a private area with a woman and interviewer and, their charts were reviewed. The data was collected by six BSc midwives and supervised by two MSc midwives who are working out of the selected public hospitals.

### Data quality control

The data was collected by trained data collectors and pretesting of the instrument was done before the actual data collection. The data collectors and supervisors were trained for one day by the investigators on how to use the data collection tool before embarking on data collection. The questionnaire was pre-tested on 5% (24 adult and 12 advanced) aged mothers who had childbirth at Zewditu Hospital, to assess the reliability, clarity, sequence, consistency, understandability, and total time it takes to finish the questionnaire. Necessary modifications and corrections were done to standardize and ensure its reliability and validity based on the results of the pre-test. The Cronbach’s alpha scores were > 0.75 for all dimensions of the tool. Daily supervision was done for data completeness by the supervisors and investigator.

### Data processing and statistical analysis

The collected data were exported from the ODK Collect Android application to SPSS version 26.0 for analysis. Descriptive statistics, including frequency tables, figures, and summaries, were used to describe the study variables. Independent t-tests were used to compare continuous outcome variables between groups and estimate mean differences. Binary logistic regression was employed to assess overall ANOs and each specific ANO after verifying logistic regression assumptions. Variables with a *p*-value < 0.20 in bivariate analysis were entered into multivariable logistic regression models to estimate adjusted odds ratios (AOR). Variables with 95% confidence intervals and *p*-values < 0.05 were considered statistically significant. The variance inflation factor (VIF) was used to assess multicollinearity, and all VIF values were found to be less than 5. In addition, standard errors of the regression coefficients were examined and found to be within acceptable ranges, suggesting that collinearity was not a concern among the independent variables. Model goodness-of-fit was evaluated using the Hosmer-Lemeshow test, and all models satisfied the assumptions (*p*-value > 0.05).

## Results

### Socio-demographic characteristics

A total of 707 mothers participated in this study, including 471 adult-aged and 236 advanced-aged mothers, with a response rate of 98.6%. The mean age of adult-aged mothers was 27.46 years (SD = 3.94), compared to 38.81 years (SD = 3.64) for AAMs. The majority of adult-aged mothers, 250 (88.7%), and 390 (84.8%) of AAMs were urban residents. Among AAMs, 227 (98.3%) were married, while 421 (91.5%) of adult-aged mothers were married. Nearly one-third (32.1%) of AAMs had a primary educational level, whereas 143 (31.1%) of adult-aged mothers had a secondary educational level. More than half of both groups were housewives (53.1% of AAMs and 54.3% of adult-aged mothers). Regarding husbands’ education, over one-third had a diploma or higher (35.7% for AAMs and 43.7% for adult-aged mothers). About one-quarter (25.6%) of AAMs’ husbands and more than one-third (35.4%) of adult-aged mothers’ husbands were government employees. In terms of family income, 150 (64.9%) AAMs and 247 (53.7%) adult-aged mothers reported an average monthly income of 5,221–13,920 Ethiopian birr (US$ 90–240), with no statistically significant difference between the groups (Table 1 and Table S1).

**Table 1:** Socio-demographic characteristics of adult-aged and advanced-aged mothers at public hospitals in addis Ababa, Ethiopia (*n* = 691)

Characteristics		Maternal age		*P*-value
		Advanced (*n* = 231)	Adult (*n* = 460)	
Variables	Category	Frequency (%)	Frequency (%)	
Residency	Urban Rural	205 (88.7) 26 (11.3)	390 (84.8) 70 (15.2)	0.155
Marital status	Married Single Divorced	227 (98.3) 3 (1.3) 1 (0.4)	421 (91.5) 35 (7.6) 4 (0.9)	0.002
Maternal educational level	Had no formal education Primary education Secondary education Diploma and above	58 (25.1) 74 (32.1) 47 (20.3) 52 (22.5)	80 (17.4) 109 (23.7) 143 (31.1) 128 (27.8)	0.001
Husband’s educational level (*n* = 648)	Had no formal education Primary education Secondary education Diploma and above	34 (15.0) 47 (20.7) 65 (28.6) 81 (35.7)	50 (11.9) 71 (16.8) 116 (27.6) 184 (43.7)	0.197
Maternal occupation	Housewife Government employee Private employee Merchant Others^a^	123 (53.2) 38 (16.5) 23 (10.0) 43 (18.6) 4 (1.7)	252 (54.8) 75 (16.3) 58 (12.6) 31 (11.1) 24 (5.2)	0.017
Husband occupation (*n* = 648)	Government employee Private employee Merchants Car driver Farmer Others^b^	58 (25.6) 55 (24.2) 52 (22.9) 41 (18.1) 15 (6.6) 6 (2.6)	149 (35.4) 88 (20.9) 98 (23.3) 47 (11.2) 20 (4.8) 19 (4.5)	0.027
Average monthly family income in ETB*	*≤* 5220 5221–13,920 *≥* 13,921	35 (15.2) 150 (64.9) 46 (19.9)	107 (23.3) 247 (53.7) 106 (23.0)	0.011

^a^(student, and day laborer), ^b^(day laborer, student, and carpenter), *US$1 = 58 Ethiopian birr (ETB) at the time of the data collection period.

### Lifestyle and medical history-related factors

Among the mothers, 40 (17.3%) AAMs and 51 (11.1%) adult-aged mothers reported alcohol consumption during their current pregnancy; of these, the majority drank alcohol occasionally (35 [87.5%] of AAMs and 37 [72.5%] of adult-aged mothers). A history of medical problems prior to the current pregnancy was significantly higher among AAMs (73 [33.3%]) compared to adult-aged mothers (71 [15.4%]). Among AAMs, 26 (11.3%) had anemia and 23 (10.0%) had chronic hypertension, whereas 25 (5.4%) and 16 (3.5%) of adult-aged mothers had these conditions, respectively. MUAC < 23 cm was more common among adult-aged mothers (142 [30.9%]) than AAMs (51 [22.1%]). Hemoglobin levels < 11 g/dl were observed in 48 (20.3%) AAMs and 105 (22.8%) adult-aged mothers. No mothers reported smoking cigarettes (Table 2).

**Table 2:** Lifestyle and medical-related factors of adult-aged and advanced-aged mothers at public hospitals in addis Ababa, Ethiopia (*n* = 691)

Characteristics			Maternal age		
			Advanced (*n* = 231)	Adult (*n* = 460)	
Variables		Category	Frequency (%)	Frequency (%)	*P*-value
Alcohol drinking		Yes No	40 (17.3) 191 (82.7)	51 (11.1) 409 (88.9)	0.022
How often did you drink alcohol (*n* = 91)		Sometimes Daily Weekly	35 (87.5) 4 (10.0) 1 (2.5)	37 (72.5) 11 (21.6) 3 (5.9)	0.219
Cigarette smoking		No	231 (100.0)	460 (100.0)	n/a
Medical problems before pregnancy		Yes No	77 (33.3) 154 (66.7)	71 (15.4) 389 (84.6)	0.001
Types of medical disease (multiple responses were possible)	Hypertension	Yes No	23 (10.0) 208 (90.0)	16 (3.5) 444 (96.5)	0.001
	DM	Yes No	9 (3.9) 222 (96.1)	5 (1.1) 455 (98.9)	0.013
	Chronic renal disease	Yes No	10 (4.3) 221 (95.7)	13 (2.8) 447 (97.2)	0.299
	Anemia	Yes No	26 (11.3) 205 (88.7)	25 (5.4) 435 (94.6)	0.006
	Peptic ulcer disease	Yes No	20 (8.7) 211 (91.3)	16 (3.5) 444 (96.5)	0.004
	HIV	Yes No	10 (4.3) 221 (95.7)	13 (2.8) 447 (97.2)	0.299
	Goiter	Yes No	3 (1.3) 228 (98.7)	3 (0.7) 457 (99.3)	0.388
	Asthma	Yes No	5 (2.2 226 (97.8)	2 (0.4) 458 (99.6)	0.032
Mothers MUAC		< 23 cm *≥* 23 cm	51 (22.1) 180 (77.9)	142 (30.9) 318 (69.1)	0.015
Maternal Rh status		Rh negative Rh positive	24 (10.4) 207 (89.6)	56 (12.2) 404 (87.8)	0.489
HIV status		Positive Negative	13 (5.6) 218 (94.4)	18 (3.9) 443 (96.1)	0.304
Hemoglobin level		< 11 g/dl *≥* 11 g/dl	47 (20.3) 184 (79.7)	105 (22.8) 355 (77.2)	0.458

### Antepartum obstetrics characteristics

More than half of AAMs (54.5%) were grand-multigravida, compared to 52.4% of adult-aged mothers who were multigravida. The mean number of gravidities was higher among AAMs (mean difference = 2.67 [2.42–2.91], *p* < 0.001). Regarding parity, 55.0% of AAMs were multipara, while 45.0% of adult-aged mothers were nulliparous (mean difference = 2.39 [2.17–2.61], *p* < 0.001). The mean inter-pregnancy interval was 34.93 ± 12.65 months for AAMs and 29.63 ± 10.61 months for adult-aged mothers; short intervals were more common among adult-aged mothers (22.6% vs. 15.7%, mean difference = 5.30 [3.22–7.38], *p* < 0.001).

A history of BOH was reported in 18.9% of AAMs and 12.8% of adult-aged mothers. Among AAMs, stillbirth was most common (56.1%), while neonatal loss predominated in adult-aged mothers (52.9%). Planned pregnancies occurred in 74.0% of AAMs and 76.7% of adult-aged mothers. All mothers had at least one ANC contact; 32.5% of AAMs and 37.2% of adult-aged mothers-initiated ANC at ≤ 16 weeks. The mean GA at first ANC contact (17.90 ± 4.54 vs. 17.35 ± 4.90 weeks) and mean number of ANC contacts (6.33 ± 2.07 vs. 6.17 ± 1.89) did not differ significantly.

Vaccination coverage was high: TT at least once (90.9% AAMs, 92.4% adult-aged) and twice (69.9% vs. 70.1%). IFAS uptake was 86.6% for AAMs and 88.7% for adult-aged mothers; significantly more AAMs took IFAS for > 3 months (40.5% vs. 31.1%). Pregnancy complications were higher among AAMs (49.4% vs. 27.6%), with PROM, APH, and pre-eclampsia more common in AAMs (21.6%, 16.9%, 12.1%) than adult-aged mothers (9.6%, 8.9%, 5.9%). Husband accompaniment to ANC was reported in 47.6% of AAMs and 50.4% of adult-aged mothers (Table 3 & Table S1).

**Table 3:** Antepartum obstetric characteristics of adult-aged and advanced-aged mothers at public hospitals in addis Ababa, Ethiopia (*n* = 691)

Characteristics			Maternal age		*P*-value
			Advanced (*n* = 231)	Adult (*n* = 460)	
Variables		Category	Frequency (%)	Frequency (%)	
Gravidity		Primigravida Multigravida Grand multigravida	14 (6.1) 91 (39.4) 126 (54.5)	195 (42.4) 241 (52.4) 24 (5.2)	0.001
Parity		Nullipara Primipara Multipara Grand multipara	15 (6.5) 24 (10.4) 127 (55.0) 65 (28.1)	207 (45.0) 127 (27.6) 120 (26.1) 6 (1.3)	0.001
Inter-pregnancy interval (*n* = 482)		< 24 months *≥* 24 months	34 (15.7) 183 (84.3)	60 (22.6) 205 (77.4)	0.055
History of BOH (*n* = 482)		Yes No	41 (18.9) 176 (81.1)	34 (12.8) 231 (87.2)	0.068
Types of BOH (*n* = 75)		Early neonatal loss Stillbirth Recurrent abortion	14 (34.1) 23 (56.1) 4 (9.8)	18 (52.9) 14 (41.2) 2 (5.9)	0.256
Status of pregnancy		Planned Unplanned and wanted	171 (74.0) 60 (26.0)	353 (76.7) 107 (23.3)	0.432
History of ANC contact		Yes	231 (100.)	460 (100.0)	n/a
GA when ANC contact started		*≤* 16 weeks > 16 weeks	75 (32.5) 156 (67.5)	171 (37.2) 289 (62.8)	0.223
Number of ANC contact		1–8 contact *≥* 8 contacts	162 (70.1) 69 (29.9)	339 (73.7) 121 (26.3)	0.322
Tetanus toxoid vaccinated		Yes No	210 (90.9) 21 (9.1)	425 (92.4) 35 (7.6)	0.501
Number of TT vaccine (*n* = 635)		1 2	65 (31.0) 145 (69.0)	127 (29.9) 298 (70.1)	0.782
IFAS		Yes No	200 (86.6) 31 (13.4)	408 (88.7) 52 (11.3)	0.420
Duration of IFAS (*n* = 608)		< 3 months *≥* 3 months	119 (59.5) 81 (40.5)	281 (68.9) 127 (31.1)	0.022
Complication during pregnancy		Yes No	114 (49.4) 117 (50.6)	127 (27.6) 333 (72.4)	< 0.001
Types of complication during pregnancy (multiple responses were possible)	Pre-eclampsia	Yes No	28 (12.1) 203 (87.9)	27 (5.9) 433 (94.1)	0.004
	Gestational DM	Yes No	9 (3.9) 222 (96.1)	7 (1.5) 453 (98.5)	0.050
	APH	Yes No	39 (16.9) 192 (83.1)	41 (8.9) 419 (91.1)	0.002
	PROM	Yes No	50 (21.6) 181 (78.4)	44 (9.6) 416 (90.4)	0.001
	HEG	Yes No	9 (3.9) 222 (96.1)	15 (3.3) 445 (96.7)	0.667
	Decreasing fetal movement	Yes No	25 (10.8) 206 (89.2)	19 (4.1) 441 (95.9)	0.001
	Decreasing amniotic fluid	Yes No	7 (3.0) 224 (97.0)	9 (2.0) 451 (98.0)	0.376
Accompanied by husband to ANC at least once		Yes No	110 (47.6) 121 (52.4)	232 (50.4) 228 (49.6)	0.485

### Intrapartum obstetrics characteristics

In this study, labor onset was spontaneous in 70.5% of AAMs and 77.6% of adult-aged mothers, with augmentation in 13.5% and 14.6%, respectively. Vaginal delivery occurred in 62.3% of AAMs and 66.7% of adult-aged mothers. The main indications for C/S delivery were failed induction (22.2% AAMs vs. 27.6% adult-aged) and fetal distress (20.6% vs. 22.9%). Vertex presentation was observed in 88.7% of AAMs and 94.0% of adult-aged mothers. Labor duration < 24 h was more frequent in AAMs (90.5%) than adult-aged mothers (81.1%). The mean total labor duration was significantly shorter in AAMs (11.58 ± 6.24 h) than adult-aged mothers (15.07 ± 6.87 h, mean difference = 3.48 [CI 2.43–4.54], *p* < 0.001). Complications during labor and delivery were higher among AAMs (62.3% vs. 54.1%), with fetal distress (27.7%), PPH (16.9%), malpresentation (15.6%), and intrapartum preeclampsia (13.4%) more common, whereas prolonged labor (18.9%) predominated in adult-aged mothers (Table 4 & Table S1).

**Table 4:** Intrapartum obstetric characteristics of adult-aged and advanced-aged mothers at public hospitals in addis Ababa, Ethiopia (*n* = 691)

Characteristics			Maternal age		*P*-value
			Advanced (*n* = 231)	Adult (*n* = 460)	
Variables		Category	Frequency (%)	Frequency (%)	
Onset of labor		Spontaneous Induced Elective C/S	163 (70.5) 48 (20.8) 20 (8.7)	357 (77.6) 77 (16.7) 26 (5.7)	0.106
Augmentation (*n* = 520)		Yes No	22 (13.5) 141 (86.5)	52 (14.6) 305 (85.4)	0.746
Mode of delivery		SVD Operative VD Emergency C/S Elective C/S	144 (62.3) 24 (10.4) 43 (18.6) 20 (8.7)	307 (66.7) 48 (10.4) 79 (17.2) 26 (5.7)	0.436
Indication of C/S (*n* = 168)		Fetal distress Failed induction Planned repeated C/S Failed VBAC Malpresentation APH Macrosomia CPD Cord prolapses Severe oligohydramnios	13 (20.6) 14 (22.2) 10 (15.9) 4 (6.3) 9 (14.3) 3 (4.8) 4 (6.3) 1 (1.6) 3 (4.8) 2 (3.2)	24 (22.9) 29 (27.6) 12 (11.4) 10 (9.5) 11 (10.5) 7 (6.6) 5 (4.8) 4 (3.8) 1 (1.0) 2 (1.9)	0.757
Fetal presentation		Vertex Breech Others*	205 (88.7) 20 (8.7) 6 (2.6)	432 (94.0) 20 (4.3) 8 (1.7)	0.051
Duration of labor		*≤* 24 h > 24 h	209 (90.5) 22 (9.5)	373 (81.1) 87 (18.9)	0.001
Complications during labor and delivery		Yes No	144 (62.3) 87 (37.7)	249 (54.1) 211 (45.9)	0.040
Types of complication during labor and delivery (multiple responses were possible)	Intrapartum preeclampsia	Yes No	31 (13.4) 200 (86.6)	36 (7.8) 424 (92.2)	0.019
	Prolonged labor	Yes No	22 (9.5) 209 (90.5)	87 (18.9) 373 (81.1)	0.001
	Obstructed labor	Yes No	5 (2.2) 226 (97.8)	9 (2.0) 451 (98.0)	0.885
	Malpresentation/malposition	Yes No	36 (15.6) 195 (84.4)	28 (6.1) 432 (93.9)	0.001
	Failed induction	Yes No	14 (6.1) 217 (93.9)	29 (6.3) 431 (93.7)	0.900
	Decreasing uterine contraction	Yes No	31 (13.4) 200 (86.6)	54 (11.7) 406 (88.3)	0.526
	Fetal distress	Yes No	64 (27.7) 167 (72.3)	78 (17.0) 382 (83.0)	0.001
	Cord prolapses	Yes No	5 (2.2) 226 (97.8)	4 (0.9) 456 (99.1)	0.157
	PPH	Yes No	39 (16.9) 192 (83.1)	46 (10.0) 414 (90.0)	0.009
	Perineal tear	Yes No	6 (2.6) 225 (97.4)	19 (4.1) 441 (95.9)	0.309
	Uterine rupture	Yes No	4 (1.7) 227 (98.3)	3 (0.7) 457 (99.3)	0.181
	Retained placenta	Yes No	8 (3.5) 223 (96.5)	12 (2.6) 448 (97.4)	0.527
	Chorioamnionitis	Yes No	7 (3.0) 224 (97.0)	9 (2.0) 451 (98.0)	0.376

*face, brow, shoulder, and cord presentation

### Neonatal characteristics

Three-fourths of adult-aged mothers (75.0%) and nearly two-thirds of AAMs (65.4%) delivered at term. The mean GA at delivery was slightly lower among AAMs (37.66 ± 1.98 weeks) than adult-aged mothers (38.26 ± 1.97 weeks, mean difference = 0.6 [CI 0.29–0.91], *p* < 0.001). Female neonates accounted for 51.1% of AAMs and 51.5% of adult-aged mothers. AAMs had higher rates of LBW (27.3% vs. 19.6%) and macrosomia (10.0% vs. 5.0%). The mean birth weight was similar between groups (AAMs: 2961.90 ± 692.46 g; adult-aged: 2945.77 ± 568.34 g). Most neonates were appropriate for gestational age (AGA), though lower in AAMs (73.7% vs. 83.3%). Low 1st- and 5th-minute Apgar scores were more frequent among AAMs (27.7% and 18.2%) than adult-aged mothers (15.7% and 9.8%; mean differences = 0.66 [0.41–0.91] and 0.56 [0.22–0.88], *p* < 0.001). NICU admission occurred in 21.2% of AAMs and 15.7% of adult-aged mothers, with birth asphyxia as the leading indication (36.8% vs. 44.4%) (Table 5 & Table S1).

**Table 5:** Neonatal characteristics of adult-aged and advanced-aged mothers at public hospitals in addis Ababa, Ethiopia (*n* = 691)

Characteristics		Maternal age		*P*-value
		Advanced (*n* = 231)	Adult (*n* = 460)	
Variables	Category	Frequency (%)	Frequency (%)	
GA at delivery	28^+0day^−36^+6days^ weeks 37^+0day^−41^+6days^ weeks *≥* 42 weeks	71 (30.7) 151 (65.4) 9 (3.9)	78 (17.0) 345 (75.0) 37 (8.0)	0.001
Sex of the newborn	Female Male	118 (51.1) 113 (48.9)	237 (51.5) 223 (48.5)	0.913
Immidate neonatal death	Yes No	13 (5.6) 218 (94.4)	12 (2.6) 448 (97.4)	0.045
Cause of immediate neonatal death (*n* = 25)	Prematurity Asphyxia Congenital malformation Unknown	6 (46.2) 4 (30.8) 1 (7.7) 2 (15.4)	4 (33.3) 4 (33.3) 1 (8.3) 3 (25.0)	0.905
Birth weight of the baby	< 2500gm (LBW) 2500-4000gm (normal) *≥* 4000gm (macrosomia)	63 (27.3) 145 (62.7) 23 (10.0)	90 (19.6) 347 (75.4) 23 (5.0)	0001
Newborn weight to gestational age	SGA AGA LGA	38 (16.4) 170 (73.7) 23 (9.9)	54 (11.7) 384 (83.3) 23 (5.0)	0.007
1 st minuet APGAR score	< 7 *≥* 7	64 (27.7) 167 (72.3)	72 (15.7) 388 (84.3)	0.001
5th minute APGAR score	< 7 *≥* 7	42 (18.2) 189 (81.8)	45 (9.8) 415 (90.2)	0.002
Birth injury	Yes No	8 (3.5) 223 (96.5)	14 (3.0) 446 (7.0)	0.767
Congenital malformation	Yes No	9 (3.9) 222 (96.1)	6 (1.3) 454 (98.7)	0.027
Types of congenital malformation (*n* = 15)	Spinal Bifida Anencephaly Hydrocephalus	5 (55.6) 2 (22.2) 2 (22.2)	3 (50.0) 2 (33.3) 1 (16.7)	0.886
IUGR	Yes No	6 (2.6) 225 (97.4)	7 (1.5) 453 (98.5)	0.326
Neonatal jaundice	Yes No	12 (5.2) 219 (94.8)	16 (3.5) 444 (96.5)	0.281
Neonate admitted to NICU	Yes No	49 (21.2) 182 (78.8)	72 (15.7) 388 (84.3)	0.070
Reason of NICU admission (*n* = 121)	Prematurity LBW Asphyxia Infection Congenital malformation Jaundice Hypoglycemia Hypothermia IUGR Unable to breastfeed	7 (14.3) 5 (10.2) 18 (36.8) 2 (4.1) 2 (4.1) 1 (2.0) 6 (12.2) 2 (4.1) 3 (6.1) 3 (6.1)	10 (13.9) 6 (8.2) 32 (44.4) 4 (5.6) 1 (1.4) 1 (1.4) 7 (9.7) 4 (5.6) 3 (4.2) 4 (5.6)	0.989
General neonatal outcome	Unfavorable Favorable	119 (51.5) 112 (48.5)	185 (40.2) 275 (59.8)	0.005

### Adverse neonatal outcomes

**Fig. 3:**
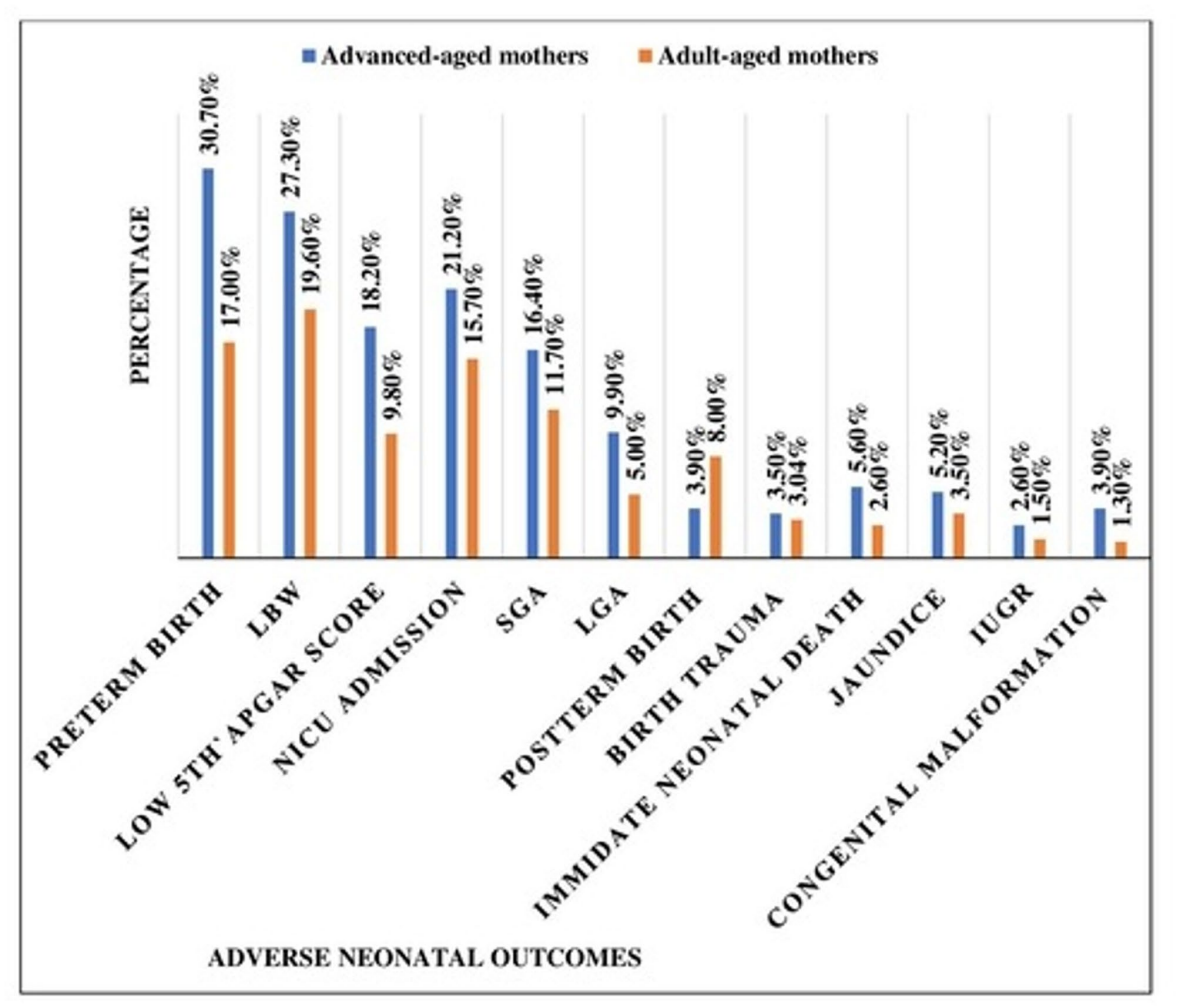
Adverse neonatal outcomes among advanced and adult-aged mothers at the public hospitals of Addis Ababa City, Ethiopia, [*n* = 691, (460 adult & 232 advanced)

The ANO scale showed acceptable reliability (Cronbach’s alpha = 0.773) across 12 items. The minimum and maximum number of ANOs was 0 and 9, respectively, with higher mean scores among AAMs (mean difference = 0.66 [0.34–0.97], *p* < 0.001) (Table S1). Overall, 51.5% (95% CI: 44.7–57.9) of AAMs and 40.2% (95% CI: 35.7–44.6) of adult-aged mothers had at least one ANO, yielding an overall prevalence of 44.0% (*n* = 304, 95% CI: 40.2–47.6). ANOs were significantly more common among AAMs, with a proportion difference of 11.3% (95% CI: 3.5–19.1, *p* = 0.05) (Fig. 3).

Specifically, AAMs had higher rates of preterm birth (13.8%, 95% CI: 0.073–0.202, *p* < 0.001), low 1 st and 5th minute Apgar scores (12.0% [95% CI: 0.058–0.183] and 8.4% [95% CI: 0.032–0.136]), LBW (7.7%, 95% CI: 0.011–0.143), LGA (4.9%, 95% CI: 0.01–0.088), immediate neonatal death (3.0%, 95% CI: 0.001–0.059), and congenital malformations (2.6%, 95% CI: 0.003–0.04). Post-term birth was higher among adult-aged mothers (4.1%, 95% CI: 0.002–0.080). There were no significant differences in SGA, NICU admission, IUGR, birth trauma, or jaundice between the two groups (Table 6).

**Table 6:** Adverse neonatal outcomes and proportion differences among adult-aged and advanced-aged mothers at public hospitals in addis Ababa, Ethiopia (*n* = 691; adult = 460, advanced-age = 231)

Characteristics		Maternal age	Frequency	Proportion	Proportion difference	*P*-value	[95% CI of difference]
Variables	Category						
Unfavorable neonatal outcome		Advanced 35^+^	119	0.515	0.113	0.005	[0.035–0.191]
		Adult (20–24)	185	0.402			
Adverse neonatal outcomes	Preterm birth	Advanced 35^+^	71	0.307	0.138	< 0.001	[0.073–0.202]
		Adult (20–24)	78	0.169			
	Post-term birth	Advanced 35^+^	9	0.039			
		Adult (20–24)	37	0.080	0.041	0.039	[0.002–0.080]
	Low-minute APGAR score	Advanced 35^+^	64	0.277	0.120	0.001	[0.058–0.183]
		Adult (20–24)	72	0.157			
	Low 5th minutes APGAR score	Advanced 35^+^	42	0.182	0.084	0.002	[0.032–0.136]
		Adult (20–24)	45	0.098			
	LBW	Advanced 35^+^	63	0.273	0.077	0.021	[0.011–0.143]
		Adult (20–24)	90	0.196			
	SGA	Advanced 35^+^	38	0.164	0.047	0.086	[0.009–0.103]
		Adult (20–24)	55	0.117			
	LGA	Advanced 35^+^	23	0.099	0.049	0.014	[0.010–0.088]
		Adult (20–24)	23	0.050			
	Birth trauma	Advanced 35^+^	8	0.034	0.004	0.772	[−0.032-0.024]
		Adult (20–24)	14	0.003			
	Immediate neonatal death	Advanced 35^+^	13	0.056	0.030	0.045	[0.001–0.059]
		Adult (20–24)	12	0.026			
	NICU admission	Advanced 35^+^	49	0.212	0.055	0.070	[0.008–0.118]
		Adult (20–24)	72	0.157			
	IUGR	Advanced 35^+^	6	0.026	0.011	0.327	[0.012–0.034]
		Adult (20–24)	7	0.015			
	Congenital malformation	Advanced 35^+^	9	0.039	0.026	0.027	[0.003–0.049]
		Adult (20–24)	6	0.013			
	Jaundice	Advanced 35^+^	12	0.052	0.017	0.281	[0.016–0.050]
		Adult (20–24)	16	0.035			

### Factors associated with adverse neonatal outcomes

Binary logistic regression was performed for overall ANOs and for each adverse outcome separately, including maternal age, socio-demographic factors, lifestyle and medical history, and antepartum and intrapartum obstetric factors as independent variables. In each bivariate analysis, variables with *P* < 0.20 were entered into the multivariable analysis, and variables with *P* < 0.05 and 95% CI not crossing one in the multivariable analysis were considered statistically significant for overall ANOs and for each adverse outcome.

After regressing the overall ANOs with maternal age and other independent variables, AAMs were 1.51 times more likely to have unfavorable neonatal outcomes compared to adult-aged mothers [AOR = 1.51, 95% CI = 1.02–2.25] (Table 7). In addition to maternal age, ANOs were higher among mothers with chronic medical problems, MUAC < 23 cm, negative maternal Rh status, short inter-pregnancy interval, not taking IFAS, pregnancy complications, cesarean delivery, and alcohol consumption (*P* < 0.05) (Table S2). Among AAMs, the likelihood of having a baby with ANOs was higher in cases of no formal education, alcohol consumption, short inter-pregnancy interval, PROM, and emergency C/S (*P* < 0.05) (Table S3). Among adult-aged mothers, the probability of having a baby with ANOs was higher in those with a monthly family income < 5,220 ETB, chronic medical problems, negative Rh status, short inter-pregnancy interval, PROM, pre-eclampsia, induced labor, or elective/emergency C/S (*P* < 0.05) (Table S4).

Likewise, each ANO was regressed with maternal age and other independent variables. AAMs were 1.84 times more likely to deliver preterm compared to adult-aged mothers [AOR = 1.84, 95% CI = 1.18–2.85]. The likelihood of having an LGA baby was 2.68 times higher among AAMs [AOR = 2.68, 95% CI = 1.31–5.49]. In contrast, the chance of having a post-term birth was 75% lower among AAMs than adult-aged mothers [AOR = 0.25, 95% CI = 0.08–0.83]. Low 5th minute Apgar score, LBW, SGA, immediate neonatal death, NICU admission, and congenital malformations were not significantly associated with maternal age in multivariable analysis (Table 7 & Tables S5–S13). Logistic regression was not performed for IUGR, birth trauma, or neonatal jaundice because their *P*-values in bivariate analysis were > 0.20.

**Table 7:** Bivariate and multivariable logistic regression analyses of adverse neonatal outcomes among adult-aged and advanced-aged mothers at public hospitals in addis Ababa, Ethiopia (*n* = 691; adult = 460, advanced-age = 231)

General neonatal outcome^a^					
Age group	Unfavorable	Favorable	COR (95% CI)	AOR (95% CI)	*P*-value
* ≥* 35 years 20–34 years	119 185	112 275	1.58 (1.15–2.17) 1	1.51 (1.02–2.25) 1	0.039*
Preterm birth^b^					
Age group	Yes	No	COR (95% CI)	AOR (95% CI)	*P*-value
* ≥* 35 years 20–34 years	71 78	160 382	2.17 (1.50–3.15) 1	1.84 (1.18–2.85) 1	0.007*
Post-term birth^c^					
Age group	Yes	No	COR (95% CI)	AOR (95% CI)	*P*-value
* ≥* 35 years 20–34 years	9 37	222 423	0.46 (0.22–0.98) 1	0.25 (0.08–0.83)	0.024*
Low fifth-minute Apgar score^d^					
Age group	Yes	No	COR (95% CI)	AOR (95% CI)	*P*-value
* ≥* 35 years 20–34 years	42 45	189 415	2.05 (1.30–3.23) 1	1.40 (0.81–2.43) 1	0.230
LBW ^e^					
Age group	Yes	No	COR (95% CI)	AOR (95% CI)	*P*-value
* ≥* 35 years 20–34 years	63 90	168 370	1.54 (1.06–2.23) 1	1.26 (0.80–2.01) 1	0.320
SGA ^f^					
Age group	Yes	No	COR (95% CI)	AOR (95% CI)	*P*-value
* ≥* 35 years 20–34 years	38 54	193 406	1.48 (0.94–2.32) 1	1.18 (0.71–1.95) 1	0.526
LGA ^g^					
Age group	Yes	No	COR (95% CI)	AOR (95% CI)	*P*-value
* ≥* 35 years 20–34 years	23 23	208 437	2.10 (1.15–3.83) 1	2.68 (1.31–5.49) 1	0.007*
Immediate neonatal death^h^					
Age group	Yes	No	COR (95% CI)	AOR (95% CI)	*P*-value
* ≥* 35 years 20–34 years	13 12	218 448	2.23 (1.01–4.96) 1	1.06 (0.33–3.36) 1	0.920
NICU admitted^i^					
Age group	Yes	No	COR (95% CI)	AOR (95% CI)	*P*-value
* ≥* 35 years 20–34 years	49 72	182 388	1.45 (0.972.17) 1	1.07 (0.61–1.89) 1	0.803
Congenital malformation^j^					
Age group	Yes	No	COR (95% CI)	AOR (95% CI)	*P*-value
* ≥* 35 years 20–34 years	9 6	222 454	3.07 (1.08–8.73) 1	2.34 (0.72–7.61) 1	0.156

^a^The dependent variable was regressed with maternal age, residency, maternal education, maternal occupation, alcohol consumption, chronic medical problems, MUAC, maternal Rh status, HIV status, Hb level, gravidity, birth interval, BOH, pregnancy status, GA at first ANC contact, number of ANC contacts, TT vaccination, IFAS, and complications during pregnancy and labor. Hosmer–Lemeshow test *P* = 0.139

^b^The dependent variable was regressed with maternal age, residency, maternal education, average monthly household income, alcohol consumption, chronic medical problems, MUAC, maternal Rh status, HIV status, Hb level, gravidity, birth interval, BOH, pregnancy status, TT vaccination, IFAS, and complications during pregnancy. Hosmer–Lemeshow test *P* = 0.319

^c^The dependent variable was regressed with maternal age, chronic medical problems, gravidity, GDM, and LGA. Hosmer–Lemeshow test *P* = 0.430

^d^The dependent variable was regressed with maternal age, residency, maternal education, average monthly household income, alcohol consumption, chronic medical problems, MUAC, maternal Rh status, Hb level, gravidity, BOH, pregnancy status, TT vaccination, IFAS, complications during pregnancy, GA at delivery, fetal presentation, complications during labor, and birth weight. Hosmer–Lemeshow test *P* = 0.360

^e^The dependent variable was regressed with maternal age, residency, maternal education, average monthly household income, alcohol consumption, chronic medical problems, MUAC, maternal Rh status, HIV status, Hb level, birth interval, BOH, pregnancy status, GA at first ANC contact, number of ANC contacts, TT vaccination, IFAS, and complications during pregnancy. Hosmer–Lemeshow test *P* = 0.798

^f^The dependent variable was regressed with maternal age, residency, average monthly household income, alcohol consumption, chronic medical problems, MUAC, maternal Rh status, HIV status, Hb level, birth interval, BOH, pregnancy status, GA at first ANC contact, number of ANC contacts, TT vaccination, IFAS, and complications during pregnancy. Hosmer–Lemeshow test *P* = 0.267

^g^The dependent variable was regressed with maternal age, maternal education, gravidity, GDM, and post-term pregnancy. Hosmer–Lemeshow test *P* = 0.756

^h^The dependent variable was regressed with maternal age, alcohol consumption, chronic medical problems, MUAC, gravidity, TT vaccination, IFAS, complications during pregnancy, preterm birth, complications during labor, and SGA. Hosmer–Lemeshow test *P* = 0.835

^i^The dependent variable was regressed with maternal age, residency, chronic medical problems, MUAC, maternal Rh status, HIV status, Hb level, birth interval, BOH, TT vaccination, complications during pregnancy, GA at delivery, fetal presentation, mode of delivery, complications during labor, and birth weight. Hosmer–Lemeshow test *P* = 0.121

^j^The dependent variable was regressed with maternal age, residency, alcohol consumption, chronic medical problems, maternal Rh status, Hb level, TT vaccination, and IFAS. Hosmer–Lemeshow test *P* = 0.289

*Significant at a *P*-value of < 0.05

## Discussion

Adverse neonatal outcomes negatively affect not only the newborn but also the mother, the family, and the wider society, contributing to substantial emotional, social, and economic burdens [53]. Most studies indicate that AMA is associated with higher rates of neonatal morbidity, including preterm birth, LBW, IUGR, and NICU admission [17–20]. However, some studies have reported no significant association between AMA and ANOs [54, 55], suggesting that the effect may vary depending on the cutoff used to define AMA. Different studies employ varying thresholds, with some using ≥ 35 years and others ≥ 40 years, which may contribute to inconsistencies in the reported risks [30]. Using a cutoff point of >35 years for AAMs, our study found that nearly half (51.5%) of AAMs and 40.2% of adult-aged mothers experienced at least one ANO. This prevalence is higher than reports from Awi Zone public hospitals, where ANOs occurred in 19.4% of cases (29.1% vs. 14.5% among AAMs and adult-aged mothers, respectively) [34], and from Jimma, where the rates were 40.5% among AAMs and 29.4% among adult-aged mothers [37]. The higher prevalence in our study may reflect differences in study settings, as public hospitals typically manage more complicated pregnancies. Previous studies also demonstrate that, in addition to maternal age, pregnancy complications substantially increase the risk of ANOs [56, 57].

In this study, the prevalence of ANOs among AAMs was lower, whereas among adult-aged mothers it was higher compared to findings from Dessie Referral Hospital, which reported 74.7% and 25.3% among advanced and adult-aged mothers, respectively [36]. This discrepancy may be explained by differences in the exposed-to-unexposed group ratio, as the Dessie study applied a 1:1 ratio, while our study used a 1:2 ratio. Another source of variation arises from outcome definitions: the Dessie study assessed adverse perinatal outcomes, including stillbirth, whereas our study excluded stillbirth, as it is not considered a component of ANOs [50]. In our study, ANOs refer only to adverse events following live births in the neonatal period; stillbirths were excluded. This differs from studies that define perinatal adverse outcomes more broadly, including stillbirth. However, the prevalence observed in our study was higher than reports from Denmark (10.8% vs. 5.4%) [11], as well as from Taiwan and Japan [58, 59]. These differences may reflect variations in socioeconomic conditions and access to quality healthcare services. Evidence further indicates that maternal nutritional status, income level, and educational attainment significantly influence pregnancy outcomes [60, 61].

Adverse neonatal outcomes were more frequent among AAMs than adult-aged mothers, with a proportion difference of 11.3% and a higher likelihood of unfavorable outcomes. This finding is consistent with studies from Ethiopia [34–39] and supported by other evidence showing that AMA significantly increases the risk of ANOs compared with younger maternal age [29, 30]. The elevated risk may be attributed to the non-modifiable nature of AMA as a determinant of ANOs [29, 34], the physiological effects of aging, and the higher likelihood of obstetric complications and pre-existing comorbidities among AAMs [20, 29, 62, 63]. Some studies have further identified rising maternal age, even without a specific cutoff, as an independent and substantial risk factor for ANOs [17–20]. Although pregnancy carries risks at all reproductive ages [64], women of AMA are particularly vulnerable. Notably, while chronic medical conditions and obstetric complications can increase the risk of ANOs in both age groups [65], older women without such comorbidities still experience worse outcomes, suggesting that AMA itself is an independent and strong risk factor [30, 66]. Moreover, the likelihood of developing maternal comorbidities during pregnancy, such as pre-eclampsia and gestational diabetes, increases with age, further heightening the risk of ANOs [63, 67].

Unfavorable neonatal outcomes were also significantly associated with several factors, including chronic medical conditions, alcohol consumption during pregnancy, maternal MUAC < 23 cm, negative maternal Rh status, short inter-pregnancy interval, failure to take IFAS, pregnancy complications, and delivery by C/S (Table S2). A similar study from Ethiopia reported that, beyond maternal age, mothers with short inter-pregnancy intervals or pregnancy complications had a higher likelihood of experiencing ANOs [34]. This highlights that, in addition to maternal age, a range of socioeconomic, obstetric, and chronic medical factors increase the risk of ANOs, both by directly affecting maternal health and indirectly influencing fetal and newborn outcomes [56, 65].

Separate analysis showed that in both groups, the risk of ANOs was higher among mothers with unfavorable socio-economic status, alcohol consumption, short inter-pregnancy interval, PROM, emergency C/S, chronic medical conditions, negative Rh status, pre-eclampsia, and induced labor (Tables S3 & S4). These findings indicate that ANO risk is influenced not only by maternal age but also by socio-demographic, medical, and obstetrical factors, consistent with studies from Ethiopia [34–36], and other countries [68–70]. Specifically, pre-eclampsia can impair uteroplacental blood flow, reducing oxygen and nutrient supply to the fetus and increasing the risk of ANOs [71]. Similarly, short inter-pregnancy intervals may not allow adequate maternal recovery, leading to uterine and placental insufficiency, compromised fetal development, and higher neonatal risk [70, 72].

Babies born to AAMs were more likely to be delivered prematurely compared to those born to adult-aged mothers. This finding is consistent with studies conducted in Ethiopia [35–38],, and other countries [68, 73, 74]. A possible explanation is that the risk of medical and obstetrical complications increases with advancing maternal age, as co-morbidities may predispose to early labor initiation or pregnancy termination [20]. Furthermore, beyond maternal age, premature birth is also significantly associated with unfavorable socio-demographic status and pregnancy-related complications (Table S5). Evidence also shows that other maternal characteristics and obstetric complications contribute to various adverse neonatal outcomes ANOs [56, 65].

On the contrary, post-term birth was significantly more common among adult-aged mothers compared to AAMs. This is consistent with previous evidence showing that advanced maternal age is less likely to be associated with post-term birth [75]. Beyond maternal age, post-mature delivery was also significantly associated with multigravidity, GDM, and having a LGA infant (Table S6). This may reflect the fact that the majority of adult-aged mothers in this study were primiparous, and primiparity is one of the strongest and most consistently reported risk factors for post-term pregnancy compared to multiparity, which is more common among AAMs [76].

Babies born to AAMs had a lower fifth-minute Apgar score, with a proportion difference of 8.4% compared to those born to adult-aged mothers. Similar findings have been reported in previous studies [36, 38]. However, after adjusting for other variables, maternal age was no longer significantly associated with low Apgar scores. Instead, low fifth-minute Apgar scores were significantly associated with gestational age at delivery, complications during labor and delivery, LBW, and alcohol consumption during pregnancy (Table S7). This is supported by other studies showing that low fifth-minute Apgar scores are influenced by maternal socio-demographic factors as well as antepartum and intrapartum obstetric characteristics [51]. Evidence indicates that complications such as prematurity or prolonged labor adversely affect neonatal outcomes, as these conditions compromise fetal oxygenation and directly impair neonatal adaptation at birth [77].

This study found that babies born to AAMs had a higher proportion of LBW, with a proportion difference of 7.7% compared to those born to adult-aged mothers. This finding is consistent with studies conducted in Ethiopia [35, 36, 38], and Finland [74]. However, after adjusting for other variables, LBW was more likely among mothers with MUAC < 23 cm, those who consumed alcohol during pregnancy, mothers with negative Rh status, hemoglobin level < 11 g/dl, a BOH, initiation of ANC contact after 16 weeks of GA, lack of IFAS intake, and the presence of pregnancy-related complications (Table S8). Similar findings have been reported in studies from Ethiopia [78, 79], and Afghanistan [80], which indicate that fetal birth weight is influenced by maternal socio-economic and obstetric factors.

Furthermore, this study revealed that babies born to AAMs were more likely to be LGA or macrosomic compared to those born to adult-aged mothers. Similar findings have been reported in studies conducted in the United Kingdom [19]. LGA was also more common among babies born to mothers with no formal education, those who developed GDM, and those who experienced post-term delivery (Table S10). Evidence indicates that post-term pregnancy is associated with an increased risk of delivering a macrosomic baby [75]. This increased risk is likely due to continued fetal growth beyond the optimal gestational period [81], which can subsequently contribute to ANOs.

The immediate neonatal mortality rate was higher among babies born to AAMs, with a proportion difference of 3.0% compared to adult-aged mothers. This may be attributed to the higher rate of labor and delivery complications among AAMs; in this study, complications occurred in 62.3% of AAMs versus 54.1% of adult-aged mothers. Studies have shown that mothers at the extremes of reproductive age have a higher risk of neonatal mortality compared to adult-aged mothers [17, 21, 82], likely reflecting the increased risk of maternal comorbidities with advancing age, which can compromise pregnancy outcomes [20, 65]. When analyzed with other variables, neonatal mortality was more common among mothers who were not vaccinated with TT, gave preterm birth, or had SGA babies (Table S11). Prematurity and weight-related factors, such as LBW and SGA, further compromise neonatal outcomes, as these infants often have difficulty adapting to extrauterine life and are more prone to hypothermia, hypoglycemia, respiratory distress, sepsis, and other complications, collectively increasing neonatal mortality rates [83]. Additionally, lack of TT vaccination during pregnancy is associated with an increased risk of neonatal deaths [84].

Congenital malformation was significantly more common among AAMs at a proportion difference of 2.6% than adult-aged mothers. This finding is supported by a systematic review and meta-analysis of observational studies, which reported that women in the older maternal age group had increased odds of having children with congenital anomalies compared with those in the 20–34-year age group [10, 11]. This might be attributed to chromosomal abnormalities, which are known to increase with advancing maternal age, thereby elevating the risk of congenital malformation, along with other environmental factors [11, 85]. However, in the present study, congenital malformations were not statistically associated with maternal age when adjusted for other factors. Instead, they were more common among mothers residing in rural areas, those who consumed alcohol, and those who did not use IFAS (Table S13). Previous studies have highlighted low socioeconomic status and alcohol consumption during gestation as significant maternal risk factors for congenital malformations [86]. Furthermore, there is robust evidence that not using IFAS is strongly associated with congenital anomalies, particularly NTDs such as spina bifida and anencephaly [87].

In this study, no significant differences were observed between AAMs and adult-aged mothers in terms of SGA, neonatal jaundice, NICU admission, birth trauma, or IUGR. These findings contrast with previous studies, which reported higher rates of SGA [67], NICU admission [30], IUGR [33], and neonatal jaundice [73], among infants born to AAMs. Adult-aged mothers are often considered the lowest-risk group, experiencing fewer obstetric complications and lower rates of neonatal trauma compared to mothers at the extremes of maternal age. In the present study, outcomes such as SGA and NICU admission were more common among newborns of mothers with MUAC < 23 cm, HIV-positive status, a history of adverse obstetric outcomes, non-use of IFAS IFAS, pregnancy complications, preterm or post-term birth, cesarean delivery, LBW, or macrosomia (Tables S9 & S12). These findings suggest that, beyond maternal age, neonatal outcomes are strongly influenced by unfavorable socio-demographic conditions, maternal comorbidities, and obstetric factors. This conclusion is supported by evidence from other studies [56, 65].

### Limitations of the study

This study has several limitations. Only immediate and first 24-hour neonatal outcomes were assessed, so longer-term effects were not captured. The exclusion of home, health center, and private facility births may limit the generalizability of the findings. Recall bias could have affected some reproductive variables, such as GA at ANC initiation, number of ANC contacts, TT vaccination, and IFAS duration. The cross-sectional, hospital-based design prevents causal inference, while unmeasured factors such as socioeconomic status, maternal nutrition, and lifestyle may have influenced outcomes. With respect to AMA, residual confounding by parity, fertility history, or assisted reproduction could not be excluded. The categorization of AMA as ≥ 35 years may have obscured differences between subgroups (e.g., 35–39 vs. ≥40 years). Selection bias is also possible, as older women who conceive and deliver in hospitals may represent a healthier subgroup with better access to care than the general population. Finally, the study may have been underpowered to detect associations for rare outcomes such as congenital malformations and neonatal death.

## Conclusions and recommendations

In this study, ANOs were higher among advanced-age mothers compared to adult-aged mothers, particularly preterm birth, LGA, LBW, low fifth-minute Apgar score, immediate neonatal death, and congenital malformations, while SGA, NICU admission, IUGR, birth trauma, and jaundice did not differ significantly. Beyond maternal age, ANOs were influenced by socio-demographic, medical, and obstetric factors, including rural residence, low education, low income, chronic medical conditions, short inter-pregnancy interval, malnutrition, multigravidity, bad obstetric history, alcohol use, HIV positivity, anemia, non-use of IFAS, late ANC initiation, lack of TT vaccination, pregnancy complications, and cesarean or complicated deliveries.

These findings highlight the need to monitor and manage pregnancies among older mothers. Strategies to reduce ANOs include promoting optimal childbearing age through family planning and education, strengthening maternal healthcare services to ensure early ANC, vaccination, and management of complications, providing additional support to high-risk mothers, and conducting longitudinal studies to assess neonatal outcomes beyond the first 24 h, including home and private facility births. Additionally, increasing women’s access to education, comprehensive maternal health services, and awareness of the benefits of early adulthood pregnancies can help reduce ANOs in the general population. Healthcare providers should deliver evidence-based counseling, provide preventive care such as TT vaccination and IFAS, and offer extra care to high-risk mothers to detect and manage complications early. Furthermore, future research should focus on prospective longitudinal studies to evaluate the long-term impact of AMA on neonatal outcomes beyond the first 24 h, including during the late neonatal period, infancy, and early childhood development.

## Supplementary Information

Supplementary Material 1.

Supplementary Material 2.

## Data Availability

All related data has been presented within the manuscript. The data set supporting the conclusion of this article is available from the corresponding author upon reasonable request.

## Abbreviations

AAMs: Advanced Aged Mothers
AGA: Appropriate for Gestational Age
AMA: Advanced Maternal Age
ANC: Antenatal Care
ANOs: Adverse Neonatal Outcomes
AOR: Adjusted Odds Ratio
APH: Ante-Ppartum Hemorrhage
CI: Confidence Interval
COR: Crude Odds Ratio
C/S: Cesarean Section
BOH: Bad Obstetric History
DHS: Demographic Health Survey
FP: Family Planning
GA: Gestational Age
GDM: Gestational Diabetes Mellitus
GMH: Gandhi Memorial Hospital
IFAS: Iron and Folic Acid Supplmentation
IUGR: Intra-Uterine Growth Restriction
LBW: Low Birth Weight
LGA: Large for Gestational Age
MCH: Maternal and Child Health
MSH: Minilik Specialize Hospital
MUAC: Mid-Upper Arm Circumference
NICU: Neonatal Intensive Care Unit
ODK: Open Data Kit
PROM: Premature Rupture of Membranes
PsH: Petros Hospital
SD: Standard Deviation
SDGs: Sustainable Developmental Goals
SGA: Small for Gestational Age
SPH: Saint Paulo’s Hospital
TASH: Tikur Anbasea Specialized Hospital
Y12H: Yekatit 12 Hospital
TT: Tetanus Toxoid
