# Supplementary material for: Advanced maternal age increases the risk of adverse neonatal outcomes: a comparative study in Ethiopia
Source: BMC Pregnancy Childbirth. 2025 Oct 28;25:1144. doi: 10.1186/s12884-025-08316-2 (PMC12570516; doi:10.1186/s12884-025-08316-2)
Supplement: Supplementary file 1 — Supplementary Material 1. [file 12884_2025_8316_MOESM1_ESM.docx]

**ANNEXES**

**Annex I. Information sheet**

**Title of the research:** Comparison of the adverse neonatal outcomes and their associated factors among women with adult and advanced-aged pregnancy at the public hospitals of Addis Ababa City, Ethiopia, 2024: Hospital-based comparative cross-sectional study

**Name of the organization**: Addis Ababa University, College of Health Sciences

**Name of the sponsor**: Saint Paulo’s Hospital

**Name of principal investigator**: Yonas Mengistu Abebe

Email address: yonasmengistu@gmail.com

**Purpose of the research project**

The main aim of this research project is to compare the adverse neonatal outcomes and their associated factors among women with adult and advanced-aged pregnancies at the public hospitals of Addis Ababa City, Ethiopia. Assessing the status of neonatal adverse outcomes helps to design appropriate intervention programs to address the problem and to take appropriate actions to reduce the problems

**Study period**- March 1/2024 to March 30/2024

**Process of the study**

Permission processed from Addis Ababa University for administrators of the respective public hospitals of Addis Ababa city. The study involves women who utilize labor and delivery services in Addis Ababa city public hospitals. You are selected to be one of the study participants if you are willing to take part in this study and we kindly invite you to take part in our project. If you are willing to participate, we are so happy and we need you to clearly understand the aim of this study and show your agreement. Finally, you are kindly requested to give your genuine response.

**Risk and/or discomfort**:

There is no risk or discomfort that you will face by participating in this research except dedication of time (a maximum of 30 minutes) for responding. Any personal information registered in registration books will not be copied and transferred to other bodies. Every piece of information will be kept confidential.

**Benefits**

Your participation is important to know the magnitude of adverse neonatal outcomes among women with adult and advanced maternal ages. However, you have no risk or direct benefit in participating in this research project.

**Incentives/payments for participating**

You will not be provided with any incentives or payment to take part in this project.

**Confidentiality**

The information collected from you will be kept confidential and stored in a file, without your name by assigning a code number to it, and no report of the study ever identifies you.

**Right to refuse or withdraw**

You have a full right to refuse to participate in this research. You have also a full right to withdraw from this study at any time you wish.

**Annex II. Consent form**

Comparison of the adverse neonatal outcomes and its associated factors among women with adult and advanced aged pregnancy at the public hospitals of Addis Ababa City, Ethiopia, 2024

Dear!

My name is _______________. I am working as a data collector in a study conducted by Yonas Mengistu at selected public hospitals in Adds Ababa City. The research supported in collaboration with Addis Ababa University College of Health Sciences, Department of Midwifery, and Saint Paulo's Hospital. I have identified you as a study participant hoping that you would be willing to help me by providing some information. As part of this study, different questions are prepared to be completed by you. For unclear questions, if you need clarification you can ask at any time. Since your participation in this survey depends on your voluntary basis you have the full right to refuse, to participate, and to stop at any time. I would like to ask you a few questions about your socio-demographic characteristics, reproductive history, health service utilization, and the health of your newborn baby, which may take 25 to 30 minutes. Certainly, I assure you that your name or your newborn baby's name will not be recorded anywhere. The confidentiality of the information you provided to me will be maintained and won't be accessed by a third party, but it's used for research only and burnt by the end of the survey. Your role in the success of the research is important and I appreciate your contribution to the research. You have a full right to refuse part or the whole questionnaires and no one enforces you to do so. However, your honest participation and answers to the questionnaire will help us in a better understanding of the problem and give guidance on how to intervene in the study area.

If you have any questions regarding this study, you can contact the principal investigator **Yonas** **Mengistu Abebe:** email address: [yonasmengistu@gmail.com](mailto:yonasmengistu@gmail.com)**.** Even you can call for institutional review board with phone number ____________

So, are you willing to participate actively and honestly? I understand the advantage of the research, and the roles I will have in the research and have agreed to participate in the research. (*If yes, let her sign and go ahead, if No stop here*.)

Yes Signature of the participant _____________ No

Questionnaire code _____________

Signature of data collector: _____________________ Date: __________________________

Signature of data supervisors: _____________________ Date: _______________________

**Annex III: English version Questionnaire**

**Part –I. Socio-demographic characteristics**

| **S.No** | **Variables** | **Response** | **Skip to** |
| --- | --- | --- | --- |
|  | Age | ______________ in complete years |  |
|  | Residence | 1. Urban 2. Rural |  |
|  | Marital status | 1. Married 2. Unmarried 3. Divorced 4. Widowed |  |
|  | Maternal educational level | 1. Unable to read and write 2. Can read and write 3. Primary education 4. Secondary education 5. Diploma and above |  |
|  | Maternal occupation | 1. Housewife 2. Government employee 3. Private employee 4. Merchant 5. Other, specify ________ |  |
|  | Husband educational level | 1. Unable to read and write 2. Can read and write 3. Primary education 4. Secondary education 5. Diploma and above |  |
|  | Husband occupation | 1. Government employee 2. Private employee 3. Merchant 4. Other, specify ________ |  |
|  | Average household monthly income | _______________ ETB |  |

**Part-II. Lifestyle and medical history-related factors**

| **S.No** | **Variables** | **Response** | **Skip to** |
| --- | --- | --- | --- |
|  | Did you drink alcohol during this pregnancy? | 1. Yes 2. No | If 2 skip to Q203 |
|  | If yes for Q201, how often? | 1. Sometimes (occasionally) 2. Daily 3. Weekly |  |
|  | Did you smoke cigarettes during this pregnancy | 1. Yes 2. No | If 2 skip to Q205 |
|  | Did you have medical problems before the current pregnancy? | 1. Yes 2. No | If 2 skip to Q209 |
|  | If yes for Q205---what type of pre-pregnancy disease? (more than one answer possible) | 1. Hypertension 2. Diabetic mellitus 3. Chronic renal disease 4. Anemia 5. Others, specify______ |  |
|  | Mother's middle upper arm circumference | 1. 23 cm and above 2. Less than 23 cm |  |
|  | Maternal Rh status | 1. Rh positive 2. Rh-negative 3. Unknown |  |
| 1. 2 | HIV status | 1. Negative 2. Positive 3. Unknown |  |
|  | Hemoglobin level | 1. >11g/dl 2. <11 g/dl |  |

**Part-III. Reproductive and obstetric history**

| **S.No** | | **Variables** | **Response** | **Skip to** |
| --- | --- | --- | --- | --- |
|  | Gravidity? | | ______________in number |  |
|  | Parity? | | ______________in number |  |
|  | Inter-pregnancy interval? | | _____________in months or years |  |
|  | Did you have a bad obstetric history? | | 1. Yes 2. No | If 2 skip to Q308 |
|  | If yes for Q306, what type of bad obstetric history? (More than one answer possible) | | 1. Recurrent spontaneous abortion (three or more) 2. Stillbirth 3. Early neonatal death 4. Others, specify ______ |  |
|  | What was the status of the last pregnancy? | | 1. Planned, wanted 2. Unplanned, wanted 3. Unplanned, unwanted |  |
|  | Did you attend pregnancy checkups/ANC for this pregnancy? | | 1. Yes 2. No | If 2 skip to Q312 |
|  | If yes for Q308, at what gestational age did you start ANC? | | ______months  ______weeks |  |
|  | If yes for Q308, how many times did you receive ANC? | | ______in numbers  ______don’t know |  |
|  | Did you receive a tetanus injection in the last pregnancy? | | 1. Yes 2. No | If 2 skip to Q314 |
|  | If yes for Q312, during your last pregnancy, how many times did you receive tetanus injection? | | _____________ in numbers |  |
|  | Have you received Iron and folic acid supplementation During your last pregnancy? | | 1. Yes 2. No | If 2 skip to Q316 |
|  | If yes for how many months | | ________months |  |
|  | Did you have any complications during your last pregnancy? | | 1. Yes 2. No | If 2 skip to Q318 |
|  | If yes for Q. No 316 what type of complication did you get? (more than one answer possible) | | 1. Pregnancy induced hypertension (Preeclampsia/Eclampsia) 2. Gestational diabetes mellitus 3. Antepartum hemorrhage 4. Premature rupture of membranes 5. Excessive vomiting/ hyperemesis gravidarum 6. Decreasing of fetal movement 7. Decreasing of amniotic fluid 8. Others, specify __________ |  |
|  | Did your spouse/partner come to a health facility for antenatal care purposes during the last pregnancy? | | 1. Yes 2. No |  |

**Checklist template**

**Part-A. Obstetric history-related chart review/checklist questions**

| **S.No** | | **Variables** | **Response** | **Skip to** |
| --- | --- | --- | --- | --- |
|  | The onset of labor? | | 1. Spontaneous 2. Induction 3. Elective cesarean section | If 2 or 3 skip to Q103 |
|  | If the onset of the labor is spontaneous, was it augmented? | | 1. Yes 2. No |  |
|  | What was the delivery type/mode of delivery? | | 1. Spontaneous vaginal delivery 2. Elective cesarean Section 3. Emergency cesarean section 4. Vaginal operative delivery 5. Other procedures, specify _______ |  |
|  | Indication for cesarean section | | 1. Fetal distress 2. Failed VBAC 3. Planned repeated VBAC 4. Malpresentation 5. Failed induction 6. Others, specify __________ |  |
|  | What was the presentation of the fetus during delivery? | | 1. Vertex presentation 2. Breech presentation 3. Shoulder presentation 4. Face presentation 5. Other(specify) ___________ |  |
|  | Total time duration from initiation of labor to delivery? | | ________ hours |  |
|  | Did you get any complications during this labor and delivery? | | 1. Yes 2. No | If 2 skip to Part B |
|  | If yes for Q-108, what type of complication (more than one answer possible)? | | 1. Intrapartum preeclampsia/eclampsia 2. Prolonged labor 3. Obstructed labor 4. Malpresentation/malposition 5. Failed induction 6. Decreasing of uterine contraction 7. Fetal distress 8. Umbilical cord prolapses 9. Post-partum hemorrhage 10. Perianal tear 11. Uterine rupture 12. Retained placenta 13. Infections 14. Others, specify __________ |  |

**Part-B. Neonatal outcomes chart review/checklist questions**

| **S.No** | | **Variables** | **Response** | **Skip to** |
| --- | --- | --- | --- | --- |
|  | At what gestational age did she deliver this neonate? | | ________weeks of gestation |  |
|  | What is the sex of the newborn baby? | | 1. Male 2. Female |  |
|  | Birth trauma | | 1. Yes 2. No | If 1 skip to Q205 |
|  | Types of birth trauma | | 1. Cephalohematoma 2. Brachial plexus 3. Fractures of the clavicle 4. Fractures of the humerus 5. Intracranial hemorrhage 6. Others, specify ______ |  |
|  | Immediate neonatal death | | 1. Yes 2. No |  |
|  | If neonatal mortality, what was the cause? | | 1. Prematurity 2. Infection 3. Asphyxia 4. Congenital malformation 5. Others, specify___________ |  |
|  | What was the birth weight of the baby (in grams)? | | ________ grams |  |
|  | What was the weight of newborn to gestational age? | | 1. Small for gestation 2. Appropriate for gestation 3. Large for gestation |  |
|  | APGAR score 1^st^ minute after birth | | ________(write the score) |  |
|  | APGAR score 5^th^ minute after birth | | ________(write the score) |  |
|  | Does the newborn have any form of gross congenital malformation? | | 1. Yes 2. No | If 2 skip to Q213 |
|  | If yes for Q-211, what was the type/diagnosis of malformation? (more than one answer possible) | | 1. Hydrocephalus 2. Anencephaly 3. Spinal Bifida 4. Others, specify |  |
|  | Does the newborn have neonatal jaundice at birth? | | 1. Yes 2. No |  |
|  | Was the newborn admitted to the NICU? | | 1. Yes 2. No |  |
|  | If yes for Q-213, what was the reason for admission to NICU? (more than one answer possible) | | 1. Prematurity 2. LBW 3. Asphyxia 4. Infection 5. Congenital malformation 6. Jaundice 7. Hupoglacimia 8. Hypothermia 9. IUGR 10. Unable to breast feed 11. Other (specify) ___________ |  |

**The end. Thank you for your time!!!**
