# Supplementary material for: Advanced maternal age increases the risk of adverse neonatal outcomes: a comparative study in Ethiopia
Source: BMC Pregnancy Childbirth. 2025 Oct 28;25:1144. doi: 10.1186/s12884-025-08316-2 (PMC12570516; doi:10.1186/s12884-025-08316-2)
Supplement: Supplementary file 2 — Supplementary Material 2. [file 12884_2025_8316_MOESM2_ESM.docx]

**Supplementary tables**

***Table S1*:** Descriptive statistics and independent sample proportion test results for continuous socio-demographic, antepartum, intrapartum, and neonatal characteristics of advanced and adult-aged mothers at the public hospitals of Addis Ababa City, Ethiopia, (n=691)

| **Variables** | **Advanced (n=231)** | | | | **Adult (n=460)** | | | | **Advanced vs adult** | | |
| --- | --- | --- | --- | --- | --- | --- | --- | --- | --- | --- | --- |
|  | Minimum | Maximum | Mean | SD | Minimum | Maximum | Mean | SD | Mean difference | [95% CI of difference] | *P*-value |
| Maternal age in years | 35 | 50 | 38.81 | 3.64 | 20 | 34 | 27.46 | 3.94 | 11.35 | [10.74-11.96] | <0.001 |
| Average monthly family income in ETB | 1400 | 20000 | 9398.94 | 3934.48 | 1300 | 30000 | 9311.30 | 4546.42 | 87.64 | [-569.66-744.93] | 0.793 |
| Gravidity | 1 | 10 | 4.73 | 2.02 | 1 | 7 | 2.06 | 1.23 | 2.67 | [2.42-2.91] | <0.001 |
| Parity | 0 | 8 | 3.35 | 1.83 | 0 | 5 | 0.96 | 1.12 | 2.39 | [2.17-2.61] | <0.001 |
| Inter-pregnancy interval in month | 15 | 96 | 34.93 | 12.65 | 12 | 72 | 29.63 | 10.61 | 5.30 | [3.22-7.38] | <0.001 |
| GA when ANC contact started | 4 | 28 | 17.90 | 4.54 | 4 | 32 | 17.35 | 4.90 | 0.55 | [-0.19-1.29] | 0.146 |
| Number of ANC contacts | 2 | 12 | 6.33 | 2.07 | 1 | 12 | 6.17 | 1.89 | 0.16 | [-0.17-0.47] | 0.346 |
| The total duration of labor in an hour | 1 | 30 | 11.58 | 6.24 | 1 | 36 | 15.07 | 6.87 | 3.48 | [2.43-4.54] | <0.001 |
| GA at delivery | 32 | 43 | 37.66 | 1.98 | 32 | 44 | 38.26 | 1.97 | 0.60 | [0.29-0.91] | <0.001 |
| Birth weight in gram | 1200 | 4600 | 2961.90 | 692.46 | 18000 | 5000 | 2945.77 | 568.34 | 16.13 | [-87.49-119.76] | 0.760 |
| First-minute APGAR score | 1 | 9 | 6.19 | 1.96 | 0 | 9 | 6.85 | 1.34 | 0.66 | [0.41-0.91] | <0.001 |
| Fifth-minute APGAR score | 0 | 10 | 7.48 | 2.78 | 0 | 10 | 8.04 | 1.66 | 0.56 | [0.22-0.88] | <0.001 |
| Number of adverse neonatal outcomes | 0 | 9 | 1.79 | 2.31 | 0 | 9 | 1.13 | 1.78 | 0.66 | [0.34-0.97] | <0.001 |

**Table S2:** Bivariate and multivariate analyses of the general ANOs among advanced and adult-aged mothers in the public hospitals of Addis Ababa City, Ethiopia, [n=691, (n= 460 adult & advanced =231)].

| **Independent variables** | **Category** | **Neonatal outcome** | | **COR (95% CI)** | **AOR (95% CI)** | **P-value** |
| --- | --- | --- | --- | --- | --- | --- |
|  |  | **Unfavorable** | **Favorable** |  |  |  |
| Maternal age | >35  20-34 | 119  185 | 112  275 | 1.58 (1.15-2.17)  1 | 1.51 (1.02-2.25)  1 | 0.039* |
| Residency | Rural  Urban | 49  255 | 47  340 | 1.39 (0.90-2.14)  1 | 0.93 (0.54-1.61)  1 | 0.801 |
| Maternal educational level | Had no formal education  Primary education  Secondary education  Diploma and above | 77  79  78  70 | 61  104  112  110 | 1.98 (1.26-3.11)  1.19 (0.78-1.81)  1.09 (0.72-1.66)  1 | 1.90 (0.97-3.72)  1.62 (0.87-3.01)  1.25 (0.70-2.24)  1 | 0.063  0.128  0.448 |
| Maternal occupation | Housewife  Merchants  Others  Private employee  Government employee | 158  44  19  33  50 | 217  50  9  48  63 | 0.92 (0.60-1.40)  1.11 (0.64-1.92)  2.66 (1.11-6.38)  0.87 (0.49-1.54)  1 | 0.83 (0.51-1.33)  1.20 (0.64-2.23)  1.87 (0.66-5.26)  0.99 (0.52-1.87)  1 | 0.433  0.571  0.238  0.976 |
| Alcohol drinking | Yes  No | 60  244 | 31  356 | 2.82 (1.78-4.49)  1 | 2.19 (1.29-3.71)  1 | 0.003* |
| Chronic medical problems | Yes  No | 92  212 | 56  331 | 2.56 (1.76-3.73)  1 | 2.02 (1.31-3.10)  1 | 0.001* |
| MUAC | <23 cm  >23 cm | 108  196 | 85  302 | 1.96 (1.40-2.74)  1 | 1.58 (1.07-2.34)  1 | 0.021* |
| Maternal Rh status | Negative  Positive | 50  284 | 30  357 | 2.34 (1.45-3.79)  1 | 1.88 (1.09-3.23)  1 | 0.022* |
| HIV status | Positive  Negative | 18  286 | 13  372 | 1.81 (0.87-3.76)  1 | 1.46 (0.60-3.54)  1 | 0.406 |
| Hb level | <11 g/dl  >11 g/dl | 85  219 | 67  320 | 1.85 (1.29-2.67)  1 | 1.12 (0.71-1.77)  1 | 0.611 |
| Gravidity | Multigravida  Primigravida | 204  100 | 278  109 | 0.80 (0.58-1.11)  1 | 0.70 (0.04-12.29)  1 | 0.806 |
| Inter-pregnancy interval | <24 months  >=24 months | 53  151 | 41  237 | 2.03 (1.29-3.20  1 | 1.98 ( (1.19-3.31)  1 | 0.009* |
| BOH | Yes  No | 42  262 | 33  354 | 1.72 (1.06-2.79)  1 | 1.60 (0.91-2.80)  1 | 0.104 |
| Status of the pregnancy | Unplanned  Planned | 82  220 | 83  304 | 1.40 (0.98-1.98)  1 | 1.18 (0.78-1.79)  1 | 0.433 |
| GA at first ANC contact | >16 weeks  <16 weeks | 210  94 | 235  152 | 1.44 (1.05-1.99) | 1.20 (0.81-1.80)  1 | 0.366 |
| Number of ANC contact | < 8 contacts  >8 contacts | 236  68 | 265  122 | 1.60 (1.13-2.26)  1 | 1.44 (0.97-2.13)  1 | 0.068 |
| TT vaccinated | No  Yes | 37  267 | 19  368 | 2.68 (1.51-4.77)  1 | 1.43 (0.70-2.93)  1 | 0.327 |
| IFAS | No  Yes | 60  244 | 23  364 | 3.89 (2.34-6.46)  1 | 2.86 (1.62-5.02) | <0.001* |
| Complication during pregnancy | Yes  No | 151  153 | 90  297 | 3.26 (2.35-4.51)  1 | 2.77 (1.92-4.00) | <0.001* |
| Onset of labor | Elective C/S  Induced  Spontaneous | 25  73  206 | 21  52  314 | 1.81 (0.99-3.33)  2.14 (1.44-3.18)  1 | 1.05 (0.48-2.28)  1.56 (0.98-2.48)  1 | 0.909  0.060 |
| Mode of delivery | C/S  Vaginal | 92  212 | 76  311 | 1.78 (1.25-2.52)  1 | 2.07 (1.39-3.08)  1 | <0.001* |
| Complication during labor and delivery | Yes  No | 194  110 | 199  189 | 1.67 (1.23-2.27)  1 | 1.08 (0.73-1.59)  1 | 0.699 |

*Significant at a P-value of <0.05. Hosmer and Lemeshow test P-value =0.139

**Table S3:** Bivariate and multivariate analyses of ANOs among advanced aged mothers in the public hospitals of Addis Ababa City, Ethiopia, (n=231).

| **Independent variables** | **Category** | **Neonatal outcome** | | **COR (95% CI)** | **AOR (95% CI)** | **P-value** |
| --- | --- | --- | --- | --- | --- | --- |
|  |  | **Unfavorable** | **Favorable** |  |  |  |
| Residency | Rural  Urban | 21  98 | 5  105 | 4.59 (1.66-12.63)  1 | 0.98 (0.26-3.61)  1 | 0.973 |
| Maternal educational level | Had no formal education  Primary education  Secondary education  Diploma and above | 46  30  22  21 | 12  44  25  31 | 5.66 (2.44-13.14)  1.01 (0.49-2.07)  1.30 (0.58-2.88)  1 | 5.88 (2.09-16.52)  1.25 (0.53-2.91)  1.34 (0.54-3.32)  1 | <0.001*  0.612  0.524 |
| Average household monthly income | <5220  5221-13920  >13920 | 22  76  21 | 13  74  25 | 2.01 (0.82-4.95)  1.22 (0.63-2.37)  1 | 0.74 (0.21-2.68)  1.05 (0.45-2.43)  1 | 0.650  0.908 |
| Alcohol drinking | Yes  No | 34  85 | 6  106 | 7.07 (2.83-17.62)  1 | 4.72 (1.65-13.50)  1 | 0.004* |
| Chronic medical problem | Yes  No | 48  71 | 29  83 | 1.93 (1.11-3.38)  1 | 0.89 (0.41-1.94)  1 | 0.778 |
| MUAC | <23 cm  >23 cm | 34  85 | 17  95 | 2.23 (1.16-4.29)  1 | 1.85 (0.82-4.17)  1 | 0.141 |
| Maternal Rh status | Negative  Positive | 17  102 | 7  105 | 2.50 (0.99-6.28)  1 | 1.42 (0.45-4.53)  1 | 0.550 |
| Hb level | <11 g/dl  >11 g/dl | 32  87 | 15  97 | 2.38 (1.21-4.69)  1 | 1.41 (0.52-3.81)  1 | 0.493 |
| Inter-pregnancy interval | <24 months  >=24 months | 24  89 | 10  94 | 2.53 (1.15-5.60)  1 | 4.26 (1.72-10.53)  1 | 0.002* |
| BOH | Yes  No | 27  92 | 14  98 | 2.05 (1.01-4.16)  1 | 1.39 (0.57-3.37)  1 | 0.466 |
| Status of pregnancy | Unplanned  Planned | 38  81 | 22  90 | 1.92 (1.05-3.51)  1 | 1.22 (0.59-2.56)  1 | 0.590 |
| GA at first ANC contact | >16 weeks  <16 weeks | 86  33 | 70  42 | 1.56 (0.90-2.72)  1 | 1.31 (0.66-2.62)  1 | 0.438 |
| Number of ANC contact | < 8 contacts  >8 contacts | 88  31 | 74  38 | 1.46 (0.83-2.570  1 | 1.04 (0.48-2.35)  1 | 0.926 |
| TT vaccinated | No  Yes | 16  103 | 5  107 | 2.32 (1.17-9.40)  1 | 2.38 (0.67-8.47)  1 | 0.180 |
| APH | Yes  No | 24  95 | 15  97 | 1.63 (0.81-3.30)  1 | 0.77 (0.32-1.87)  1 | 0.568 |
| PROM | Yes  No | 38  81 | 12  100 | 3.91 (1.92-7.97)  1 | 4.36 (1.94-9.82)  1 | <0.001* |
| Preeclampsia | Yes  No | 18  101 | 10  102 | 1.82 (0.80-4.13)  1 | 1.49 (0.53-4.17) | 0.446 |
| Mode of delivery | Emergency C/S  Elective C/S  Operative VD  SVD | 26  10  6  77 | 17  10  18  67 | 1.33 (0.66-2.66)  0.87 (0.34-2.22)  0.29 (0.11-0.77)  1 | 2.35 (1.04-5.35)  1.92 (0.67-5.48)  0.46 (0.15-1.39)  1 | 0.041*  0.225  0.171 |
| Complication during labor and delivery | Yes  No | 80  39 | 64  48 | 1.54 (0.90-2.63)  1 | 1.63 (0.80-3.30)  1 | 0.176 |

*Significant at a P-value of <0.05. Hosmer and Lemeshow test P-value =0.290

**Table S4:** Bivariate and multivariate analyses of ANOs among adult-aged mothers in the public hospitals of Addis Ababa City, Ethiopia, (n= 460].

| **Independent variables** | **Category** | **Neonatal outcome** | | **COR (95% CI)** | **AOR (95% CI)** | **P-value** |
| --- | --- | --- | --- | --- | --- | --- |
|  |  | **Unfavorable** | **Favorable** |  |  |  |
| Maternal occupation | Housewife  Merchants  Others  Private employee  Government employee | 89  24  16  24  32 | 163  27  8  34  43 | 1.63 (0.89-2.99)  3.66 (1.51-8.89)  1.29 (0.72-2.320  1.36 (0.81-2.300  1 | 0.71 (0.38-1.33)  1.54 (0.67-3.56)  2.34 (0.75-7.30)  1.13 (0.51-2.51)  1 | 0.287  0.311  0.144  0.760 |
| Average household monthly income | <5220  5221-13920  >13920 | 57  94  34 | 50  153  72 | 2.41 (1.38-4.21)  1.30 (0.80-2.11)  1 | 2.98 (1.52-5.84)  1.56 (0.88-2.74)  1 | 0.001*  0.125 |
| Alcohol drinking | Yes  No | 26  159 | 25  250 | 1.63 (0.91-2.93)  1 | 0.94 (0.45-1.98) | 0.880 |
| Chronic medical problem | Yes  No | 44  141 | 27  248 | 2.87 (1.70-4.83)  1 | 4.16 (2.24-7.72)  1 | <0.001* |
| MUAC | <23 cm  >23 cm | 74  111 | 68  207 | 2.03 (1.36-3.03)  1 | 1.47 (0.92-2.34)  1 | 0.107 |
| Maternal Rh status | Negative  Positive | 33  152 | 23  252 | 2.38 (1.35-4.20)  1 | 2.42 (1.25-4.67)  1 | 0.009* |
| HIV status | Positive  Negative | 10  175 | 8  267 | 1.91 (0.74-4.93)  1 | 1.49 (0.46-4.86)  1 | 0.510 |
| Hb level | <11 g/dl  >11 g/dl | 53  132 | 52  223 | 1.72 (1.11-2.67)  1 | 1.09 (0.64-1.86)  1 | 0.758 |
| Gravidity | Grand multigravida  Multigravida  Primigravida | 11  80  94 | 13  161  101 | 0.91 (0.39-2.13)  0.53 (0.36-0.79)  1 | 0.80 (0.03-22.43)  0.69 (0.03-16.43)  1 | 0.896  0.818 |
| Inter-pregnancy interval | <24 months  >24 months | 29  62 | 31  143 | 2.16 (1.20-3.88)  1 | 2.04 (1.04-4.01)  1 | 0.039* |
| GA at first ANC contact | >16 weeks  <16 weeks | 124  61 | 165  110 | 1.36 (0.92-2.01)  1 | 1.07 (0.65-1.77)  1 | 0.787 |
| Number of ANC contact | < 8 contacts  >8 contacts | 148  37 | 191  84 | 1.76 (1.13-2.74)  1 | 1.59 (0.95-2.67)  1 | 0.080 |
| TT vaccinated | No  Yes | 21  164 | 14  261 | 2.39 (1.18-4.83)  1 | 1.36 (0.56-3.32)  1 | 0.496 |
| IFAS | Yes  No | 33  152 | 19  256 | 2.92 (1.61-5.32)  1 | 1.82 (0.87-3.78)  1 | 0.109 |
| PROM | Yes  No | 22  163 | 22  253 | 1.55 (0.83-2.89)  1 | 2.19 (1.02-4.70)  1 | 0.043* |
| Preeclampsia | Yes  No | 19  166 | 8  267 | 3.82 (1.63-8.92)  1 | 6.33 (2.38-16.83)  1 | <0.001* |
| Onset of labor | Elective C/S  Induced  Spontaneous | 15  45  125 | 11  32  232 | 2.53 (1.13-5.68)  2.61 (1.58-4.31)  1 | 3.10 (1.23-7.80)  2.45 (1.30-4.62)  1 | 0.016*  0.006* |
| Mode of delivery | Emergency C/S  Elective C/S  Operative VD  SVD | 41  15  16  113 | 38  11  32  194 | 1.85 (1.12-3.05)  2.34 (1.04-5.27)  0.86 (0.45-1.63)  1 | 2.04 (1.08-3.82)  -  0.54 (0.25-1.19)  1 | 0.027*  -  0.126 |
| Fetal presentation | Malpresentation  Vertex | 18  167 | 10  265 | 2.86 (1.29-6.34)  1 | 2.01 (0.79-5.07)  1 | 0.141 |
| Complication during labor and delivery | Yes  No | 11  71 | 35  140 | 1.66 (1.14-2.43)  1 | 0.77 (0.45-1.31)  1 | 0.333 |

*Significant at a P-value of <0.05. Hosmer and Lemeshow test P-value =0.754

**Table S5:** Bivariate and multivariate analyses of preterm birth among advanced and adult-aged mothers in the public hospitals of Addis Ababa City, Ethiopia, [n=691, (n= 460 adult & advanced =231)].

| **Independent variables** | **Category** | **Preterm birth** | | **COR (95% CI)** | **AOR (95% CI)** | **P-value** |
| --- | --- | --- | --- | --- | --- | --- |
|  |  | **Yes** | **No** |  |  |  |
| Maternal age | >35  20-34 | 71  78 | 160  382 | 2.17 (1.50-3.15)  1 | 1.84 (1.18-2.85)  1 | 0.007* |
| Residency | Rural  Urban | 21  122 | 69  473 | 1.51 (0.93-2.47)  1 | 0.88 (0.49-1.60)  1 | 0.686 |
| Maternal educational level | Had no formal education  Primary education  Secondary education  Diploma and above | 40  42  38  29 | 98  141  152  151 | 2.12 (1.24-3.65)  1.55 (0.92-2.62)  1.30 (0.76-2.22)  1 | 0.91 (0.45-1.84)  1.15 (0.64-2.07)  1.05 (0.58-1.89)  1 | 0.795  0.648  0.874 |
| Average household monthly income | <5220  5221-13920  >13920 | 39  87  23 | 103  310  129 | 2.12 (1.19-3.78)  1.57 (0.95-2.60)  1 | 1.92 (1.02-3.59)  1.38 (0.81-2.34)  1 | 0.042*  0.240 |
| Alcohol drinking | Yes  No | 38  111 | 53  489 | 3.16 (1.98-5.03)  1 | 2.34 (1.39-3.94)  1 | 0.001* |
| Chronic medical problem | Yes  No | 41  108 | 107  435 | 1.64 (1.02-2.34)  1 | 0.79 (0.47-1.32)  1 | 0.363 |
| MUAC | <23 cm  >23 cm | 57  92 | 136  406 | 1.85 (126-2.71)  1 | 1.66 (1.07-2.58)  1 | 0.023* |
| Maternal Rh status | Negative  Positive | 34  115 | 46  496 | 3.19 (1.96-5.19)  1 | 2.74 (1.61-4.68)  1 | <0.001* |
| HIV status | Positive  Negative | 12  137 | 19  523 | 2.41 (1.14-5.09)  1 | 2.04 (0.90-4.65)  1 | 0.089 |
| Hb level | <11 g/dl  >11 g/dl | 44  109 | 108  434 | 1.68 (1.12-2.54) | 1.06 (0.63-1.79) | 0.824 |
| Gravidity | Grand multigravida  Multigravida  Primigravida | 54  60  35 | 96  272  174 | 2.80 (1.71-4.58)  1.10 (0.69-1.73)  1 | 1.45 (0.03-69.76)  0.99 (0.02-45.77)  1 | 0.850  0.997 |
| Inter-pregnancy interval | <24 months  >=24 months | 30  84 | 64  304 | 1.70 (1.03-2.78)  1 | 1.67 (0.96-2.91)  1 | 0.068 |
| BOH | Yes  No | 25  124 | 50  492 | 1.98 (1.18-3.33)  1 | 1.43 (0.78-2.61)  1 | 0.242 |
| Status of pregnancy | Unplanned  Planned | 47  102 | 120  422 | 1.62 (1.09-2.42)  1 | 1.36 (0.86-2.15)  1 | 0.186 |
| TT vaccinated | No  Yes | 20  129 | 36  506 | 2.18 (1.22-3.89)  1 | 1.23 (0.58-2.60)  1 | 0.593 |
| IFAS | No  Yes | 35  114 | 48  494 | 3.16 (1.95-5.11)  1 | 2.36 (1.37-4.08)  1 | 0.002* |
| Complication during pregnancy | Yes  No | 76  73 | 165  377 | 2.38 (1.64-3.44)  1 | 1.76 (1.17-2.64)  1 | <0.001* |

*Significant at a P-value of <0.05. Hosmer and Lemeshow test P-value =0.319

**Table S6:** Bivariate and multivariate analyses of post-term birth among advanced and adult-aged mothers in the public hospitals of Addis Ababa City, Ethiopia, [n=691, (n= 460 adult & advanced =231)].

| **Independent variables** | **Category** | **Post-term birth** | | **COR (95% CI)** | **AOR (95% CI)** | **P-value** |
| --- | --- | --- | --- | --- | --- | --- |
|  |  | **Yes** | **No** |  |  |  |
| Maternal age | >35  20-34 | 9  37 | 222  423 | 0.46 (0.22-0.98)  1 | 0.25 (1.08-0.83) | 0.024* |
| Chronic medical problem | Yes  No | 14  32 | 134  511 | 1.67 (0.87-3.22)  1 | 1.38 (0.59-3.20)  1 | 0.457 |
| Gravidity | Grand multigravida  Multigravida  Primigravida | 9  11  26 | 141  321  183 | 0.45 (0.20-0.99)  0.24 (0.12-0.50)  1 | 0.52 (0.15-1.77)  0.22 (0.09-0.49)  1 | 0.293  <0.001* |
| GDM | Yes  No | 7  39 | 9  636 | 12.68 (4.49-35.86)  1 | 11.89 (3.13-45.19)  1 | <0.001* |
| LGA | Yes  No | 18  28 | 28  617 | 14.17 (7.01-28.61)  1 | 19.55 (8.28-46.16)  1 | <0.001* |

*Significant at a P-value of <0.05. Hosmer and Lemeshow test P-value =0.430

**Table S7:** Bivariate and multivariate analyses of low fifth minute Apgar score among advanced and adult-aged mothers in the public hospitals of Addis Ababa City, Ethiopia, [n=691, (n= 460 adult & advanced =231)].

| **Independent variables** | **Category** | **Low 5^th^ minute APGAR** | | **COR (95% CI)** | **AOR (95% CI)** | **P-value** |
| --- | --- | --- | --- | --- | --- | --- |
|  |  | **Asphyxiated** | **Non-asphyxiated** |  |  |  |
| Maternal age | >35  20-34 | 42  45 | 189  415 | 2.05 (1.30-3.23)  1 | 1.40 (0.81-2.43)  1 | 0.230 |
| Residency | Rural  Urban | 23  64 | 73  531 | 2.61 (1.53-4.47)  1 | 1.89 (0.97-3.66)  1 | 0.059 |
| Maternal educational level | Had no formal education  Primary education  Secondary education  Diploma and above | 30  21  15  21 | 108  162  175  159 | - 1. (1.14-3.86)   0.98 (0.52-1.87)  0.65 (0.32-1.30)  1 | 0.66 (0.28-1.55)  0.71 (0.33-1.50)  0.47 (0.21-1.06)  1 | 0.339  0.364  0.068 |
| Average household monthly income | <5220  5221-13920  >13920 | 24  49  14 | 118  348  138 | 2.01 (0.99-44.05)  1.39 (0.74-2.59)  1 | 1.37 (0.55-3.40)  1.25 (0.59-2.65)  1 | 0.500  0.553 |
| Alcohol drinking | Yes  No | 27  60 | 64  540 | 3.80 (2.25-6.40)  1 | 2.03 (1.07-3.83)  1 | 0.029* |
| Chronic medical problem | Yes  No | 25  62 | 123  481 | 1.58 (0.95-2.61)  1 | 0.78 (0.40-1.53)  1 | 0.479 |
| MUAC | <23 cm  >23 cm | 33  54 | 160  444 | 1.70 (1.06-2.71)  1 | 1.34 (0.69-2.60)  1 | 0.390 |
| Maternal Rh status | Negative  Positive | 19  68 | 61  543 | 2.49 (1.40-4.41)  1 | 1.39 (0.69-2.82)  1 | 0.360 |
| Hb level | <11 g/dl  >11 g/dl | 24  63 | 128  476 | 1.42 (0.85-2.36)  1 | 0.73 (0.38-1.40)  1 | 0.351 |
| Gravidity | Grand multigravida  Multigravida  Primigravida | 33  33  21 | 117  299  188 | 2.52 (1.39-4.57)  0.99 (0.55-1.76)  1 | 1.64 (0.62-4.35)  1.28 (0.61-2.69)  1 | 0.320  0.512 |
| BOH | Yes  No | 15  72 | 60  544 | 1.89 (1.02-3.50)  1 | 0.94 (0.39-2.72)  1 | 0.900 |
| Status of pregnancy | Unplanned  Planned | 27  60 | 140  464 | 1.49 (0.91-2.44)  1 | 1.29 (0.71-2.36)  1 | 0.399 |
| TT vaccinated | No  Yes | 17  70 | 39  565 | 3.52 (1.89-6.55)  1 | 2.15 (0.99-4.69)  1 | 0.053 |
| IFAS | No  Yes | 23  64 | 60  544 | 3.26 (1.89-5.62)  1 | 1.19 (0.54-2.64)  1 | 0.664 |
| Complication during pregnancy | Yes  No | 43  44 | 198  406 | 2.01 (1.27-3.15)  1 | 0.75 (0.42-1.36)  1 | 0.347 |
| GA at delivery | Preterm  Post-term  Term | 54  6  27 | 95  40  469 | 9.87 (5.92-16.47)  2.61 (1.02-6.68)  1 | 4.04 (2.08-7.82)  1.95 (0.65-5.87)  1 | <0.001*  0.233 |
| Fetal presentation | Malpresentation  Vertex | 12  75 | 42  562 | - 1. (1.08-4.25)   1 | 0.87 (0.37-2.07)  1 | 0751 |
| Complication during labor and delivery | Yes  No | 68  19 | 325  279 | 3.07 (1.80-5.24)  1 | 3.03 (1.65-5.56)  1 | <0.001* |
| Birth weight | LBW  Macrosomia  Normal | 57  6  24 | 96  40  468 | 11.58 (6.85-19.57)  2.92 (1.13-7.57)  1 | 4.27 (2.21-8.27)  1.79 (0.59-5.45)  1 | <0.001*  0.302 |

*Significant at a P-value of <0.05. Hosmer and Lemeshow test P-value =0.360

**Table S8:** Bivariate and multivariate analyses of LBW among advanced and adult-aged mothers in the public hospitals of Addis Ababa City, Ethiopia, [n=691, (n= 460 adult & advanced =231)].

| **Independent variables** | **Category** | **LBW** | | **COR (95% CI)** | **AOR (95% CI)** | **P-value** |
| --- | --- | --- | --- | --- | --- | --- |
|  |  | **Yes** | **No** |  |  |  |
| Maternal age | >35  20-34 | 63  90 | 168  370 | 1.54 (1.06-2.23)  1 | 1.26 (0.80-2.01)  1 | 0.317 |
| Residency | Rural  Urban | 30  123 | 66  472 | 1.74 (1.08-280)  1 | 1.01 (0.53-1.90)  1 | 0.981 |
| Maternal educational level | Had no formal education  Primary education  Secondary education  Diploma and above | 43  36  37  37 | 95  147  153  143 | 1.75 (1.05-2.91)  0.95 (0.57-1.58)  0.93 (0.56-1.55)  1 | 0.77 (0.42-1.47)  0.71 (0.40-1.26)  0.63 (0.35-1.13)  1 | 0.453  0.243  0.121 |
| Average household monthly income | <5220  5221-13920  >13920 | 42  85  26 | 100  312  126 | 2.03 (1.17-3.55)  1.32 (0.81-2.14)  1 | 1.51 (0.81-2.82)  1.19 (0.70-2.02)  1 | 0.196  0.527 |
| Alcohol drinking | Yes  No | 38  115 | 53  485 | 3.02 (1.90-4.81)  1 | 1.98 (1.17-3.37)  1 | 0.012* |
| Chronic medical problem | Yes  No | 47  106 | 101  437 | 1.92 (1.28-2.88)  1 | 1.01 (0.60-1.69)  1 | 0.969 |
| MUAC | <23 cm  >23 cm | 71  82 | 122  416 | 2.95 (2.03-4.30)  1 | 2.45 (1.56-3.86)  1 | <0.001* |
| Maternal Rh status | Negative  Positive | 32  121 | 48  490 | 2.70 (1.65-4.40)  1 | 2.01 (1.16-3.46)  1 | 0.012* |
| HIV status | Positive  Negative | 13  140 | 18  520 | 2.68 (1.28-5.61)  1 | 2.11 (0.89-4.99)  1 | 0.089 |
| Hb level | <11 g/dl  >11 g/dl | 56  97 | 96  442 | 2.66 (1.79-3.95)  1 | 1.66 (1.03-2.68)  1 | 0.038* |
| Inter-pregnancy interval | <24 months  >=24 months | 31  77 | 63  311 | 1.99 (1.21-3.27)  1 | 1.56 (0.89-2.74)  1 | 0.122 |
| BOH | Yes  No | 25  128 | 50  488 | 1.91 (1.14-3.20)  1 | 2.03 (1.12-3.70)  1 | 0.020* |
| Status of pregnancy | Unplanned  Planned | 45  108 | 122  416 | 1.42 (0.95-2.12)  1 | 1.11 (0.69-1.79)  1 | 0.670 |
| GA at first ANC contact | >16 weeks  <16 weeks | 110  43 | 335  203 | 1.55 (1.05-2.30)  1 | 1.58 (1.01-2.46)  1 | 0.044* |
| Number of ANC contact | < 8 contacts  >8 contacts | 121  32 | 389  158 | 1.57 (1.02-2.42)  1 | 1.09 (0.64-1.85)  1 | 0.760 |
| TT vaccinated | No  Yes | 23  130 | 33  505 | 2.71 (1.54-4.77)  1 | 1.17 (0.56-2.46)  1 | 0.676 |
| IFAS | No  Yes | 39  114 | 44  494 | 3.84 (2.38-6.19)  1 | 2.49 (1.45-4.30)  1 | 0.001* |
| Complication during pregnancy | Yes  No | 81  72 | 160  378 | 2.66 (1.84-3.84)  1 | 2.42 (1.61-3.63)  1 | <0.001* |

*Significant at a P-value of <0.05. Hosmer and Lemeshow test P-value =0.798

**Table S9:** Bivariate and multivariate analyses of SGA among advanced and adult-aged mothers in the public hospitals of Addis Ababa City, Ethiopia, [n=691, (n= 460 adult & advanced =231)].

| **Independent variables** | **Category** | **SGA** | | **COR (95% CI)** | **AOR (95% CI)** | **P-value** |
| --- | --- | --- | --- | --- | --- | --- |
|  |  | **Yes** | **No** |  |  |  |
| Maternal age | >35  20-34 | 38  54 | 193  406 | 1.48 (0.94-2.32)  1 | 1.18 (0.71-1.95)  1 | 0.526 |
| Residency | Rural  Urban | 18  74 | 78  521 | 1.62 (0.92-2.87)  1 | 0.99 (0.53-1.88)  1 | 0.936 |
| Average household monthly income | <5220  5221-13920  >13920 | 27  48  17 | 115  349  135 | 1.86 (0.97-3.59)  1.09 (0.61-1.97)  1 | 1.33 (0.65-2.37)  0.97 (0.53-1.88)  1 | 0.440  0.991 |
| Alcohol drinking | Yes  No | 20  72 | 71  528 | 2.07 (1.19-3.59)  1 | 1.09 (0.56-2.10)  1 | 0.800 |
| Chronic medical problem | Yes  No | 30  62 | 118  481 | 1.97 (1.22-3.19)  1 | 1.05 (0.58-1.89)  1 | 0.883 |
| MUAC | <23 cm  >23 cm | 45  47 | 148  451 | 2.92 (1.86-4.57)  1 | 2.48 (1.45-4.24)  1 | 0.001* |
| Maternal Rh status | Negative  Positive | 17  75 | 63  536 | 1.93 (1.07-3.47)  1 | 1.42 (0.75-2.69)  1 | 0.277 |
| HIV status | Positive  Negative | 10  82 | 21  578 | 3.36 (1.53-7.38)  1 | 2.58 (1.06-6.26)  1 | 0.036* |
| Hb level | <11 g/dl  >11 g/dl | 36  56 | 116  483 | 2.68 (1.68-4.26)  1 | 1.64 (0.94-2.87)  1 | 0.081 |
| Inter-pregnancy interval | <24 months  >=24 months | 16  46 | 78  342 | 1.52 (0.82-2.83)  1 | 0.97 (0.48-2.04)  1 | 0.970 |
| BOH | Yes  No | 17  75 | 58  541 | 2.11 (1.17-3.82)  1 | 2.40 (1.23-4.67)  1 | 0.010* |
| Status of pregnancy | Unplanned  Planned | 30  62 | 137  462 | 1.63 (1.01-2.63)  1 | 1.42 (0.85-2.37)  1 | 0.177 |
| GA at first ANC contact | >16 weeks  <16 weeks | 67  25 | 378  221 | 1.57 (0.96-2.55)  1 | 1.64 (0.96-2.81)  1 | 0.070 |
| Number of ANC contact | < 8 contacts  >8 contacts | 75  17 | 426  173 | 1.79 (1.03-3.12)  1 | 1.30 (0.68-2.48)  1 | 0.435 |
| TT vaccinated | No  Yes | 14  78 | 42  557 | 2.38 (1.24-4.56)  1 | 0.95 (0.42-2.15)  1 | 0.899 |
| IFAS | No  Yes | 24  68 | 59  540 | 3.23 (1.89-5.53)  1 | 1.29 (1.27-4.13)  1 | 0.006* |
| Complication during pregnancy | Yes  No | 51  41 | 190  409 | 2.68 (1.71-4.18)  1 | 2.42 (1.50-3.90) | <0.001* |

*Significant at a P-value of <0.05. Hosmer and Lemeshow test P-value =0.267

**Table S10:** Bivariate and multivariate analyses of LGA among advanced and adult-aged mothers in the public hospitals of Addis Ababa City, Ethiopia, [n=691, (n= 460 adult & advanced =231)].

| **Independent variables** | **Category** | **LGA** | | **COR (95% CI)** | **AOR (95% CI)** | **P-value** |
| --- | --- | --- | --- | --- | --- | --- |
|  |  | **Yes** | **No** |  |  |  |
| Maternal age | >35  20-34 | 23  23 | 208  437 | 2.10 (1.15-3.83)  1 | 2.68 (1.31-5.49)  1 | 0.007* |
| Maternal educational level | Had no formal education  Primary education  Secondary education  Diploma and above | 19  11  6  10 | 119  172  184  107 | 2.71 (1.22-6.04)  1.09 (0.45-2.63)  0.55 (0.20-1.56)  1 | 3.57 (1.43-8.87)  1.15 (0.43-3.11)  0.53 (0.17-1.65)  1 | 0.006*  0.779  0.272 |
| Gravidity | Grand multigravida  Multigravida  Primigravida | 17  18  11 | 133  314  198 | 2.30 (1.04-5.07)  1.03 (0.48-2.23)  1 | 1.53 (0.48-4.87)  1.76 (0.66-4.70)  1 | 0.473  0.262 |
| GDM | Yes  No | 6  40 | 10  635 | 9.52 (3.30-27.53)  1 | 3.76 (1.02-13.87)  1 | 0.047* |
| Post-term pregnancy | Yes  No | 18  28 | 28  617 | 14.17 (70.04-28.61)  1 | 19.85 (8.48-46.44)  1 | <0.001* |

*Significant at a P-value of <0.05. Hosmer and Lemeshow test P-value =0.756

**Table S11:** Bivariate and multivariate analyses of immediate neonatal death among advanced and adult-aged mothers in the public hospitals of Addis Ababa City, Ethiopia, [n=691, (n= 460 adult & advanced =231)].

| **Independent variables** | **Category** | **Immediate neonatal death** | | **COR (95% CI)** | **AOR (95% CI)** | **P-value** |
| --- | --- | --- | --- | --- | --- | --- |
|  |  | **Yes** | **No** |  |  |  |
| Maternal age | >35  20-34 | 13  12 | 218  448 | 2.23 (1.01-4.96) | 1.06 (0.33-3.36)  1 | 0.920 |
| Alcohol drinking | Yes  No | 9  16 | 82  584 | 4.01 (1.71-9.36)  1 | 1.85 (0.69-4.95)  1 | 0.220 |
| Chronic medical problem | Yes  No | 10  15 | 138  528 | 2.55 (1.12-5.80)  1 | 1.35 (0.51-3.58)  1 | 0.545 |
| MUAC | <23 cm  >23 cm | 12  13 | 81  485 | 2.47 (1.11-5.52)  1 | 1.53 (0.62-3.81)  1 | 0.358 |
| Gravidity | Grand multigravida  Multigravida  Primigravida | 10  10  5 | 204  322  140 | 1.26 (0.43-3.76)   - 1. (0.97-8.710   1 | 1.73 (0.48-6.30)  2.13 (0.62-7.26)  1 | 0.403  0.227 |
| TT vaccinated | No  Yes | 8  17 | 48  618 | 6.06 (2.49-14.76)  1 | 4.02 (1.51-10.68)  1 | 0.005* |
| IFAS | No  Yes | 9  16 | 74  592 | 4.50 (1.92-10.54)  1 | 1.27 (0.38-4.32) | 0.697 |
| Complication during pregnancy | Yes  No | 15  10 | 226  440 | 2.92 (1.29-6.60)  1 | 1.21 (0.44-3.35)  1 | 0.705 |
| Preterm labor | Yes  No | 17  8 | 32  534 | 8.60 (3.63-20.35)  1 | 5.68 (2.25-14.38)  1 | <0.001* |
| Complication during labor and delivery | Yes  No | 20  5 | 373  293 | 3.14 (1.16-8.47)  1 | 2.46 (0.87-6.98)  1 | 0.090 |
| SGA | Yes  No | 12  13 | 80  586 | 6.76 (2.98-15.33)  1 | 2.96 (1.17-7.43)  1 | 0.021* |

*Significant at a P-value of <0.05. Hosmer and Lemeshow test P-value =835

**Table S12:** Bivariate and multivariate analyses of NICU admission among advanced and adult-aged mothers in the public hospitals of Addis Ababa City, Ethiopia, [n=691, (n= 460 adult & advanced =231)].

| **Independent variables** | **Category** | **NICU admitted** | | **COR (95% CI)** | **AOR (95% CI)** | **P-value** |
| --- | --- | --- | --- | --- | --- | --- |
|  |  | **Asphyxiated** | **Non-asphyxiated** |  |  |  |
| Maternal age | >35  20-34 | 49  72 | 182  388 | 1.45 (0.972.17)  1 | 1.07 (0.61-1.89)  1 | 0.803 |
| Residency | Rural  Urban | 22  99 | 74  496 | 1.49 (0.88-2.51)  1 | 0.99 (0.51-1.94)  1 | 0.987 |
| Chronic medical problem | Yes  No | 36  85 | 112  458 | 1.73 (1.11-2.69)  1 | 0.86 (0.48-1.52)  1 | 0.596 |
| MUAC | <23 cm  >23 cm | 50  71 | 143  427 | 2.10 (1.40-3.16)  1 | 1.80 (1.07-3.02)  1 | 0.026* |
| Maternal Rh status | Negative  Positive | 24  97 | 56  514 | 2.27 (1.43-3.84)  1 | 1.28 (0.66-2.49)  1 | 0.465 |
| HIV status | Positive  Negative | 10  111 | 21  549 | 2.35 (1.08-5.14)  1 | 1.75 (0.63-4.89)  1 | 0.286 |
| Hb level | <11 g/dl  >11 g/dl | 37  84 | 115  455 | 1.74 (1.12-2.70)  1 | 0.95 (0.51-1.80)  1 | 0.885 |
| Inter-pregnancy interval | <24 months  >=24 months | 23  63 | 71  325 | 1.67 (0.97-2.87)  1 | 1.35 (0.68-2.67)  1 | 0.394 |
| BOH | Yes  No | 9  102 | 56  514 | 1.71 (0.97-2.99)  1 | 1.26 (0.63-2.54)  1 | 0.515 |
| TT vaccinated | No  Yes | 16  105 | 40  530 | 2.02 (1.09-3.74)  1 | 0.92 (0.42-1.99)  1 | 0.824 |
| Complication during pregnancy | Yes  No | 61  60 | 180  390 | 2.20 (1.48-3.28)  1 | 1.11 (0.66-1.84)  1 | 0.698 |
| GA at delivery | Preterm  Post-term  Term | 67  12  42 | 82  34  454 | 8.83 (5.62-13.87)  3.81 (1.84-7.92)  1 | 3.82 (2.10-6.94)  2.52 (1.07-5.94)  1 | <0.001*  0.034* |
| Mode of delivery | Emergency C/S  Elective C/S  Operative VD  SVD | 34  12  9  99 | 88  34  63  385 | 2.25 (1.40-3.62)  2.06 (1.01-4.18)  0.83 (0.39-1.76)  1 | 2.80 (1.44-5.45)  2.77 (1.14-6.75)  1.67 (0.66-4.20)  1 | 0.002*  0.025*  0.277 |
| Fetal presentation | Malpresentation  Vertex | 18  103 | 36  534 | 2.59 (1.42-4.74)  1 | 0.79 (0.34-1.85)  1 | 0.592 |
| Complication during labor and delivery | Yes  No | 89  32 | 04  266 | 2.43 (1.57-3.76)  1 | 1.78 (1.01-3.14)  1 | 0.047* |
| Birth weight | LBW  Macrosomia  Normal | 78  13  30 | 75  33  462 | 10.02 (9.84-20.06)  6.07 (2.89-12.72)  1 | 8.53 (4.71-15.46)  3.75 (1.59-8.81)  1 | <0.001*  0.002* |

*Significant at a P-value of <0.05. Hosmer and Lemeshow test P-value =0.121

**Table S13:** Bivariate and multivariate analyses of congenital malformation among advanced and adult-aged mothers in the public hospitals of Addis Ababa City, Ethiopia, [n=691, (n= 460 adult & advanced =231)].

| **Independent variables** | **Category** | **Congenital malformation** | | **COR (95% CI)** | **AOR (95% CI)** | **P-value** |
| --- | --- | --- | --- | --- | --- | --- |
|  |  | **Yes** | **No** |  |  |  |
| Maternal age | >35  20-34 | 9  6 | 222  454 | 3.07 (1.08-8.73)  1 | 2.34 (0.72-7.61)  1 | 0.156 |
| Residency | Rural  Urban | 7  8 | 89  587 | 5.77 (2.04-16.30)  1 | 3.61 (1.14-11.44)  1 | 0.029* |
| Alcohol drinking | Yes  No | 8  7 | 83  593 | 8.16 (2.89-23.10)  1 | 3.75 (1.17-12.01)  1 | 0.026* |
| Chronic medical problem | Yes  No | 7  8 | 41  535 | 3.32 (1.18-9.31)  1 | 1.67 (0.46-5.98)  1 | 0.433 |
| Maternal Rh status | Negative  Positive | 5  10 | 75  601 | 4.01 (1.33-12.04)  1 | 2.59 (0.78-8.62)  1 | 0.120 |
| Hb level | <11 g/dl  >11 g/dl | 7  8 | 145  531 | 3.20 (1.14-8.98)  1 | 1.17 (0.35-3.97)  1 | 0.795 |
| TT vaccinated | No  Yes | 6  9 | 50  626 | 8.35 (2.85-24.39)  1 | 2.11 (0.56-7.97) | 0.271 |
| IFAS | No  Yes | 10  5 | 73  603 | 16.52 (5.50-49.66)  1 | 10.87 (3.38-34.93)  1 | <0.001* |

*Significant at a P-value of <0.05. Hosmer and Lemeshow test P-value =0.289
